# Reported Adverse Effects and Attitudes among Arab Populations Following COVID-19 Vaccination: A Large-Scale Multinational Study Implementing Machine Learning Tools in Predicting Post-Vaccination Adverse Effects Based on Predisposing Factors

**DOI:** 10.3390/vaccines10030366

**Published:** 2022-02-26

**Authors:** Ma’mon M. Hatmal, Mohammad A. I. Al-Hatamleh, Amin N. Olaimat, Rohimah Mohamud, Mirna Fawaz, Elham T. Kateeb, Omar K. Alkhairy, Reema Tayyem, Mohamed Lounis, Marwan Al-Raeei, Rasheed K. Dana, Hamzeh J. Al-Ameer, Mutasem O. Taha, Khalid M. Bindayna

**Affiliations:** 1 Department of Medical Laboratory Sciences, Faculty of Applied Medical Sciences, The Hashemite University, P.O. Box 330127, Zarqa 13133, Jordan; 2 Department of Immunology, School of Medical Sciences, Universiti Sains Malaysia, Kubang Kerian, Kota Bharu 16150, Malaysia; alhatamleh@student.usm.my (M.A.I.A.-H.); rohimahm@usm.my (R.M.); 3 Department of Clinical Nutrition and Dietetics, Faculty of Applied Medical Sciences, The Hashemite University, P.O. Box 330127, Zarqa 13133, Jordan; aminolaimat@hu.edu.jo; 4 Nursing Department, Faculty of Health Sciences, Beirut Arab University, Beirut 1105, Lebanon; mirna.fawaz@bau.edu.lb; 5 Oral Health Research and Promotion Unit, Faculty of Dentistry, Al-Quds University, Jerusalem 51000, Palestine; ekateeb@staff.alquds.edu; 6 Department of Pathology and Laboratory Medicine, King Abdulaziz Medical City, Ministry of National Guard Health Affairs, P.O. Box 22490, Riyadh 11426, Saudi Arabia; alkhairyom1@ngha.med.sa; 7 King Saud bin Abdulaziz University for Health Sciences, P.O. Box 3660, Riyadh 11481, Saudi Arabia; 8 King Abdullah International Medical Research Center (KAIMRC), P.O. Box 3660, Riyadh 11481, Saudi Arabia; 9 Department of Human Nutrition, College of Health Sciences, QU Health, Qatar University, Doha P.O. Box 2713, Qatar; reema.tayyem@qu.edu.qa; 10 Department of Agro-Veterinary Science, Faculty of Natural and Life Sciences, University of Ziane Achour, BP 3117, Djelfa 17000, Algeria; lounisvet@gmail.com; 11 Faculty of Sciences, Damascus University, Damascus P.O. Box 30621, Syria; mn41@live.com; 12 Faculty of Medicine, Mansoura University, Mansoura, Dakahlia 35516, Egypt; rasheed.khd95@gmail.com; 13 Department of Biology and Biotechnology, Faculty of Science, American University of Madaba, P.O. Box 99, Madaba 17110, Jordan; h.alameer@aum.edu.jo; 14 Department of Pharmaceutical Sciences, Faculty of Pharmacy, The University of Jordan, Amman 11942, Jordan; mutasem@ju.edu.jo; 15 Department of Microbiology, Immunology and Infectious Diseases, College of Medicine and Medical Sciences, Arabian Gulf University, Manama 329, Bahrain

**Keywords:** SARS-CoV-2, nCoV-2019, coronavirus, vaccines, vaccine safety, vaccine hesitancy, adverse reactions, side effects

## Abstract

Background: The unprecedented global spread of coronavirus disease 2019 (COVID-19) has imposed huge challenges on the healthcare facilities, and impacted every aspect of life. This has led to the development of several vaccines against COVID-19 within one year. This study aimed to assess the attitudes and the side effects among Arab communities after receiving a COVID-19 vaccine and use of machine learning (ML) tools to predict post-vaccination side effects based on predisposing factors. Methods: An online-based multinational survey was carried out via social media platforms from 14 June to 31 August 2021, targeting individuals who received at least one dose of a COVID-19 vaccine from 22 Arab countries. Descriptive statistics, correlation, and chi-square tests were used to analyze the data. Moreover, extensive ML tools were utilized to predict 30 post vaccination adverse effects and their severity based on 15 predisposing factors. The importance of distinct predisposing factors in predicting particular side effects was determined using global feature importance employing gradient boost as AutoML. Results: A total of 10,064 participants from 19 Arab countries were included in this study. Around 56% were female and 59% were aged from 20 to 39 years old. A high rate of vaccine hesitancy (51%) was reported among participants. Almost 88% of the participants were vaccinated with one of three COVID-19 vaccines, including Pfizer-BioNTech (52.8%), AstraZeneca (20.7%), and Sinopharm (14.2%). About 72% of participants experienced post-vaccination side effects. This study reports statistically significant associations (*p* < 0.01) between various predisposing factors and post-vaccinations side effects. In terms of predicting post-vaccination side effects, gradient boost, random forest, and XGBoost outperformed other ML methods. The most important predisposing factors for predicting certain side effects (i.e., tiredness, fever, headache, injection site pain and swelling, myalgia, and sleepiness and laziness) were revealed to be the number of doses, gender, type of vaccine, age, and hesitancy to receive a COVID-19 vaccine. Conclusions: The reported side effects following COVID-19 vaccination among Arab populations are usually non-life-threatening; flu-like symptoms and injection site pain. Certain predisposing factors have greater weight and importance as input data in predicting post-vaccination side effects. Based on the most significant input data, ML can also be used to predict these side effects; people with certain predicted side effects may require additional medical attention, or possibly hospitalization.

## 1. Introduction

Since the first case was reported in Wuhan, China, approximately two years ago, coronavirus disease 2019 (COVID-19) is still an ongoing global pandemic caused by severe acute respiratory syndrome coronavirus 2 (SARS-CoV-2). SARS-CoV-2 is a single-stranded positive-sense RNA virus with a genome of about 30 kb, and it belongs to the *Coronaviridae* family, which is a member in the *Nidovirales* order [1]. Although the virus can disseminate to all human cells that express the angiotensin-converting enzyme 2 (ACE2) receptors, it is mainly spread from the lung, and it uses its spike proteins that bind to ACE2 to penetrate host cells [2]. Individuals with COVID-19 experienced a variety of signs and symptoms, depending on the severity of infection, that range from flu-like illness to acute respiratory distress syndrome (ARDS), with an average mortality rate of 1.8% [3].

Since the early months of the COVID-19 pandemic, the global research community has received urgent calls for the development of effective and safe vaccines, as mass vaccination is the ideal protocol and best hope for tackling viral infection [4,5]. In response, the collaboration of researchers, industry and funding bodies led to the development of several COVID-19 vaccines that were authorized and made available for use worldwide. This lightning-fast, extraordinary achievement was accompanied by a flurry of rumors and conspiracy theories about these vaccines and the virus itself, which increased the rate of vaccine hesitancy worldwide [6,7]. Although the authorized vaccines against COVID-19 have proven to be effective and safe [8], similar to any therapeutics, they may have some side effects. Studies showed that these side effects were most commonly mild and tolerable (non-life-threatening), resulted from the desired immune response, while the common side effects were flu-like symptoms and injection site pain [9,10,11,12,13]. However, COVID-19 vaccine hesitancy and acceptance rates, as well as the post-vaccination side effects, may vary according to different factors, including type of vaccine and the subjective nature and sociodemographic variables [14].

The Arab countries (also called the Arab world), 22 countries in the Middle East and North Africa (MENA) region with a population of more than 436 million population [15], are highly affected by the COVID-19 pandemic. As of 15 February 2022, the Arab countries recorded approximately 12.4 million confirmed COVID-19 cases and 162,500 deaths, but these numbers tend to be much lower than the actual numbers, due to limited testing and challenges in the attribution of the cause of death [16]. Furthermore, approximately 73.5 million (around 17%) people in the Arab population had received at least one dose of a COVID-19 vaccine by 31 August 2021 [16]. Recent studies have reported high rates of COVID-19 vaccine hesitancy among Arabs, and these rates were relatively higher than the global rate [17,18,19]. The low public acceptability of COVID-19 vaccines is most probably associated with rumors and conspiracy theories [20]. Reports from several Arab countries have shown that fear of serious post-vaccination side effects and misinformation about COVID-19 vaccines are the biggest obstacles facing mass vaccination campaigns [18,19,21,22,23,24,25,26,27,28,29,30,31,32,33,34]. Hence, this could result in delaying vaccination and waning immunity against SARS-CoV-2 over time, after initial infection or vaccination [35], and subsequently, it may prolong the burden of the COVID-19 pandemic in the region. In contrast, good knowledge about the vaccines and their side effects were significantly associated with vaccination acceptance rate [36]. Therefore, refuting the rumors and conspiracy beliefs, by enriching knowledge about the COVID-19 vaccines and their real side effects, could strengthen public confidence in COVID-19 vaccines. Thus, conducting an independent multinational study which includes most of the countries in the region to report the vaccination experience of the Arab populations is a crucial milestone to support mass COVID-19 vaccination campaigns in the region.

On the other hand, recent years have witnessed a runaway increase in the involvement of promising machine learning (ML) approaches in the field of medicine, from basic medical sciences research, to clinical decision-making [37,38]. Several studies have employed a variety of potential ML algorithms for the understanding of the nature of SARS-CoV-2 and its transmission dynamics [39,40], forecasting pandemic scenarios [41,42], predicting COVID-19 diagnosis and prognosis [43], drug repurposing [44], and vaccine development against COVID-19 [45], as well as for predicting COVID-19 vaccination willingness [46] and post-vaccination side effects [13]. Interestingly, for the post-vaccination stage, a few studies have utilized ML applications to build predictive models for the reactogenicity and morbidity incidences, and for the severity of side effects following COVID-19 vaccination [13,47]. Hence, ML approaches may help in reducing the pressure on healthcare systems by identifying red flags that may lead to reduce the hospital admissions and improve the diagnosis, prognosis, and treatment strategies. Therefore, the present study aimed to assess the side effects and perceptions among Arab populations following COVID-19 vaccination, as well as using different ML tools to predict the post-vaccination side effects based on predisposing factors.

## 2. Materials and Methods

### 2.1. Study Design and Participants

A randomized, self-reported, large-scale cross-sectional online survey was carried out from 14 June to 31 August 2021. Participants who received at least one dose of a COVID-19 vaccine from a total of 22 Arab countries were invited to be involved in this study by answering a Google Form-based questionnaire. There were no restrictions on age, gender, education, job, or socioeconomic level, as well as type of COVID-19 vaccine and date of vaccination. The survey link was circulated via social media platforms (i.e., Facebook, Instagram, and WhatsApp) and email with adequate information about the study. The study was conducted after reviewing and approving the Institutional Review Board (IRB) committee at The Hashemite University (protocol code: 3/10/2020/2021), and a consent form was obtained from each participant prior to recruitment.

### 2.2. Survey Instrument

Since Arabic is a majority and official language of the native populations in the target countries, the validated Arabic version of a questionnaire which has already been developed to assess the side effects and perceptions following COVID-19 vaccination in Jordan [13] was used, with slight modifications made to fit the Arab population context (the modified English version is attached as Supplementary Material). The survey tool was sent to a panel of experts for review and validation, and they provided overall positive feedback with slight modifications, which were then reflected on the survey. Only two new questions have been added to collect further information, including interval between receiving a COVID-19 vaccine and participating in this study, and time of COVID-19 vaccine breakthrough infection.

In the present study, the self-reported severity of post-vaccination side effects was used to evaluate the overall intensity of side effects from participants’ perspective, which is totally different from the serious adverse events that need to be clinically diagnosed, such as anaphylaxis, thrombocytopenia, myocarditis and Guillain–Barre syndrome. The overall severity was recorded generally because it was difficult to record the severity of each side effect separately. Herein, the participants share their experiences and beliefs towards post-vaccination side effects without providing any clinical data. For example, both fever and headache are not serious adverse effects, but some participants reported that they experienced these symptoms at a severe level. For many reasons, people may suffer from non-life-threatening side effects, but at a severe level which makes them feel exhausted and causes severe pain; nevertheless, they do not require hospitalization or medical interventions, or they may be unable to access health services.

### 2.3. Sample Size

According to data obtained from “Our World in Data (OWID)”, approximately 73.5 million people in Arab countries had received at least one dose of a COVID-19 vaccine by 31 August 2021 [16]. The minimum representative sample size of 664 was determined using the Raosoft online sample size calculator [48], with a 5% margin of error, a 99% confidence interval, and a 50% response distribution. As of 3 August 2021, and after reading the study aims, instructions, and accepting to participate in this survey, a total of 10,128 respondents answered the questionnaire, which represents almost 15-fold of the required sample size, and indicates that a convenience sample was used.

### 2.4. Statistical Analysis

The Microsoft Excel version 2013 (Microsoft Corporation, Redmond, WA, USA) was used to analyze the data; frequencies and percentages were measured and used as descriptive statistics, and a correlation test was performed to assess the potential correlations between predisposing factors. The statistical associations of predisposing factors with post-vaccination side effects and the overall severity were examined using the chi-square test (χ^2^) via KNIME Analytics Platform version 4.1.3 (KNIME AG, Zurich, Switzerland). In order to obtain the most significant associations, the association was considered statistically significant if *p*-value was ≤0.01.

The included predisposing factors were: gender; age; education level; being a healthcare worker; country; suffering from chronic diseases; being a smoker; suffering from food and/or drug allergies; experiencing COVID-19 infection before receiving any vaccine dose; experiencing COVID-19 vaccine hesitancy and related fears before vaccination; type of COVID-19 vaccine; interval between receiving a COVID-19 vaccine and participating in this study; number of doses; experiencing COVID-19 vaccine breakthrough infection; and time of breakthrough infection.

The included post-vaccination side effects were: tiredness; anxiety, depression and sleep disorders; fever; headache; haziness or lack-of-clarity in eyesight; injection site pain and swelling; joint pain; swollen ankles and feet; myalgia; nausea; abdominal pain; diarrhea; vomiting; bruises on the body; bleeding gums; nosebleed; chills; itchy skin or irritation and allergic reactions; sweating for no reason; cold, numbness and tingling in limbs; dizziness; clogged nose; runny nose; dyspnea; chest pain; sleepiness and laziness; irregular heartbeats; abnormal blood pressure; sore or dry throat; and cough.

### 2.5. ML Prediction

With the aim of predicting post-vaccination side effects and their overall severity (output) based on predisposing factors (input), several ML models were built based on different algorithms using KNIME Analytics Platform version 4.1.3 (KNIME AG, Zurich, Switzerland). The used ML tools, their principles and settings, as well as evaluation tools, are summarized in Table 1.

## 3. Results

### 3.1. Participant Demographics

Of 10,128 respondents, a total of 10,064 were included in this study; the other respondents (*n* = 64) were excluded due to providing inconsistent answers or incomplete responses (missing entries). The participated individuals were from 19 countries of the Arab world, almost 44% (*n* = 4466) were male and 56% (*n* = 5598) were female, and the majority (59%, *n* = 5892) were 20 to 39 years old. Furthermore, almost 89% of the participants were studying or completed their undergraduate (63%, *n* = 6337) or postgraduate (26%, *n* = 2608) studies, while 2975 (30%) were healthcare workers. Further characteristics of participants are shown in Table 2.

### 3.2. Health-Related Information

**Figure 1:**
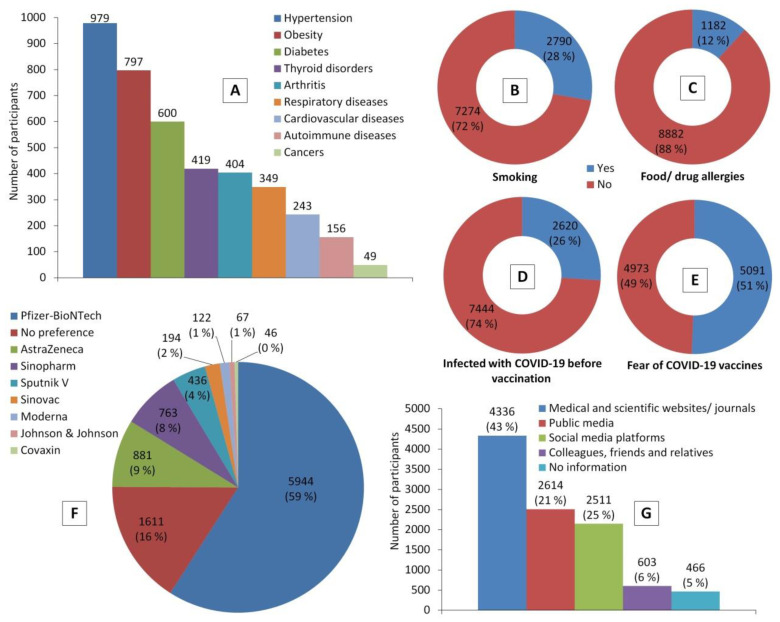
Participants’ health status indicators and their perceptions towards COVID-19 vaccines before receiving a COVID-19 vaccine. Chart (**A**) represents the most common chronic diseases that were reported by participants. (**B**–**E**) show proportions of participants who are smokers, have food and/or drug allergies, had experienced COVID-19 infection, had experienced COVID-19 vaccine hesitancy and related fears, respectively. (**F**) shows frequencies of COVID-19 vaccines preferred by participants, while (**G**) shows the credible sources of information about COVID-19 vaccines among them.

Chronic diseases were reported by around 28% of the participants (*n* = 2799). The most common diseases were hypertension, obesity, and diabetes; 10% (*n* = 979), 8% (*n* = 797), and 6% (*n* = 600), respectively. Besides, almost 28% of the participants (*n* = 2790) were smokers, 12% (*n* = 1182) had food and/or drug allergies, and 26% (*n* = 2620) experienced a lab-confirmed COVID-19 infection. Before COVID-19 vaccination, almost half of the participants (*n* = 5091) experienced COVID-19 vaccine hesitancy and related fears and anxiety, despite reporting their trust in the credibility of medical/scientific websites and journals as a source of information about COVID-19 vaccines (*n* = 4336, 43%). Moreover, Pfizer-BioNTech (officially called Comirnaty) was the highly preferred vaccine (*n* = 5944, 59%) by participants compared to other types of COVID-19 vaccines (Figure 1).

### 3.3. Vaccination Information

The findings showed that almost 88% (*n* = 8830) of the participants were vaccinated against COVID-19 with one of three types of vaccines, including the Pfizer-BioNTech, AstraZeneca and Sinopharm vaccines, 52.8% (*n* = 5310), 20.7% (*n* = 2087), and 14.2% (*n* = 1433), respectively. Regardless of vaccine type, the proportions of the participants who received single (*n* = 5356, 53%) and two (*n* = 4708, 47%) doses were relatively close to some extent, respectively. However, it is clearly shown that beyond these close proportions, the largest proportion of those who received the Sinopharm and Moderna vaccines successfully completed their second shots, 64% (*n* = 922) and 71% (*n* = 86), respectively (Table 3).

### 3.4. Post-Vaccination Information

**Figure 2:**
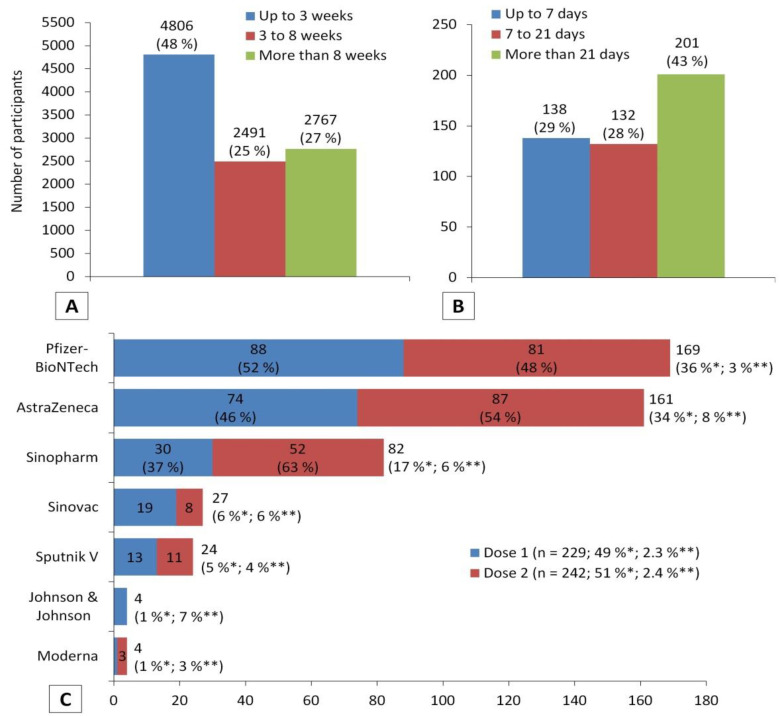
Participants’ post-vaccination information. (**A**) Interval between receiving a COVID-19 vaccine and participating in this study (*n* = 10,064). (**B**) Time of COVID-19 vaccine breakthrough infection (*n* = 471). (**C**) Characterization of participants who experienced COVID-19 vaccine breakthrough infections based on the type of vaccine and number of doses (*n* = 471; 4.7%). * The perception was calculated out of the total number of participants who experienced vaccine breakthrough infection (*n* = 471); ** the perception was calculated out of the total number of participants who received the vaccine (Table 3).

During enrolment in the study, 4806 (48%) of the participants were still in the first three weeks after COVID-19 vaccination, and 2491 (25%) were between the 3rd and 8th week, while 2767 (27%) were more than two months after. In addition, a total of 471 (4.7%) participants experienced a COVID-19 vaccine breakthrough infection after different periods of time that were classified into three categories: up to one week of receiving a COVID-19 vaccine (*n* = 138, 29%), one to three weeks (*n* = 132, 28%), and more than three weeks (*n* = 201, 43%). The proportion of infected participants with COVID-19 after vaccination was different based on the type of vaccine. In general, out of the total number of participants with breakthrough infection (*n* = 471), the largest proportion was for those who received the Pfizer-BioNTech vaccine (*n* = 169, 36%), which is the most common vaccine in the present study. However, this number counts for only 3% of the total number of participants who received the Pfizer-BioNTech vaccine (*n* = 5310), which is the smallest proportion compared to other vaccines. The largest proportion of participants with breakthrough infection was among participants who received the AstraZeneca vaccine (8%). The proportions of breakthrough COVID-19 infection among participants who received a single dose and two doses were relatively close, 2.3% (*n* = 229) and 2.4% (*n* = 242), respectively (Figure 2).

**Figure 3:**
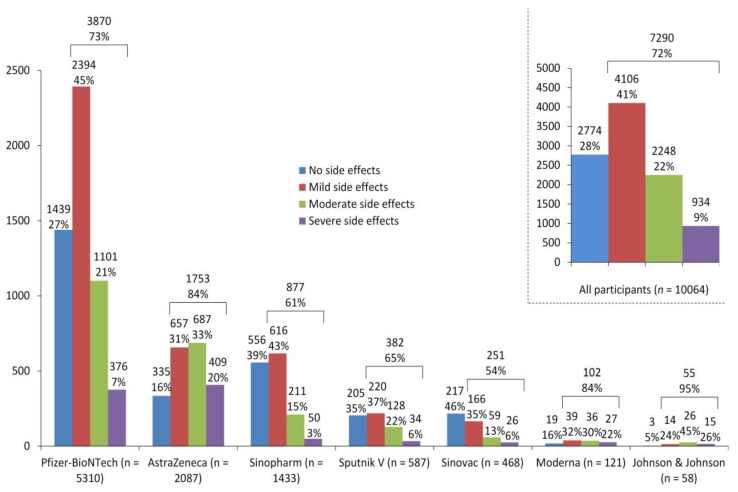
Severity of side effects following COVID-19 vaccination.

Following COVID-19 vaccination, almost 28% (*n* = 2774) of the participants did not experience any side effects, while about 41% (*n* = 4106) and 22% (*n* = 2248) of participants reported mild and moderate side effects, respectively. Only 9% (*n* = 934) suffered from severe side effects. Nevertheless, these proportions varied according to the type of vaccines. For example, 20% of participants who received the AstraZeneca vaccine suffered from severe side effects, compared to 7% and 3% for the Pfizer-BioNTech and Sinopharm vaccines, respectively. Further details are shown in Figure 3.

**Figure 4:**
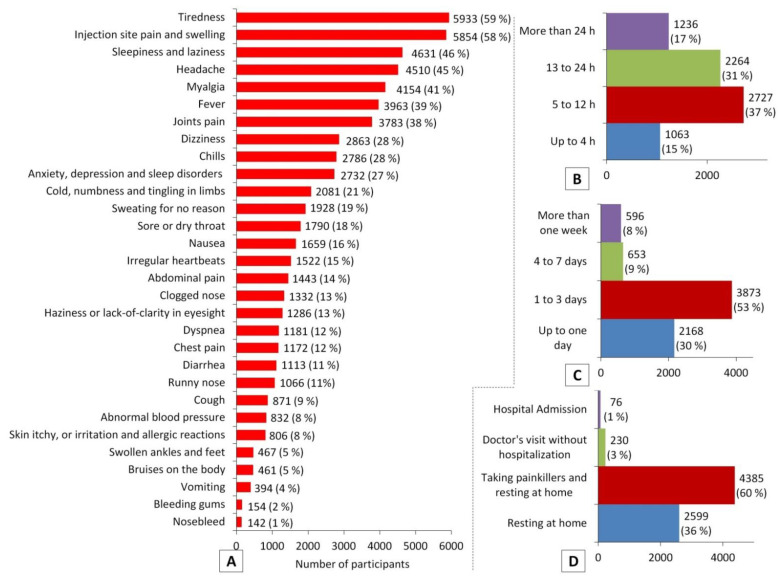
Side effects of COVID-19 vaccines. (**A**), the most common post-vaccination side effects; (**B**), interval between receiving a COVID-19 vaccine and experiencing side effects; (**C**), duration of post-vaccination side effects; (**D**), coping responses to post-vaccination side effects.

Among 7290 (72%) of the participants who experienced post-vaccination side effects, the most common side effects were tiredness (59%), injection site pain and swelling (58%), sleepiness and laziness (46%), headache (45%), myalgia (41%), fever (39%), joint pain (38%), dizziness (28%), chills (28%), anxiety and sleep disorders (27%), and numbness and tingling in limbs (21%). Most participants (83%) experienced post-vaccination side effects during the 24 h after receiving a COVID-19 vaccine, while only 17% (*n* = 1236) experienced these after more than 24 h. The post-vaccination side effects lasted for up to three days, as reported by 83% of participants, and for up to 24 h among 30% of them. Although resting at home, with or without taking painkillers, was enough for the majority of participants (96%, *n* = 6984) to overcome these side effects, 4% of participants suffered from severe side effects that required a doctor’s intervention—3% (*n* = 230)—or even hospital admission—1% (*n* = 76) (Figure 4).

### 3.5. Participants’ Perceptions

**Figure 5:**
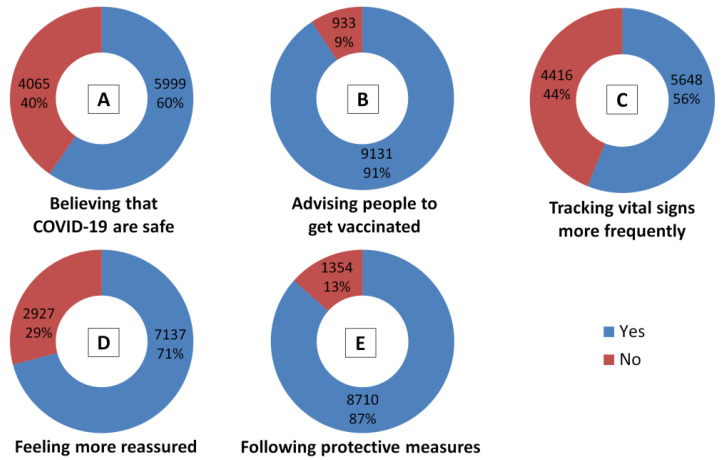
Participants’ responses to belief-based questions after COVID-19 vaccination.

Based on their COVID-19 vaccination experience, the participants were asked to express their own attitudes towards the COVID-19 vaccines by answering specific questions. More than half of participants (60%, *n* = 5999) believe in the long-term safety of the COVID-19 vaccines. The majority (91%, *n* = 9131) advised people to get vaccinated against COVID-19. Almost 56% (*n* = 5648) noticed that they track their vital signs more than usual to determine any abnormalities post-vaccination, while 71% (*n* = 7137) felt much more reassured. Lastly, most participants (87%, *n* = 8710) believed that even those who have been vaccinated for COVID-19 still need to wear a mask, practice social distancing and wash their hands frequently, as well as any other applicable mandatory safety measures, health standards and regulations to prevent/control COVID-19 (Figure 5).

### 3.6. Association of Predisposing Factors and Post-Vaccination Side Effects

The χ^2^ test showed that there were significant associations (*p* < 0.01) between the gender and age of participants and the frequencies of all post-vaccination side effects, except bleeding gums and nosebleeds (*p* > 0.01). There were statistically significant differences (*p* < 0.01) between healthcare workers and other workers in the frequencies of the following post-vaccination side effects: fever; haziness or lack-of-clarity in eyesight; swollen ankles and feet; abdominal pain; diarrhea; itchy skin, or irritation and allergic reactions; sweating for no reason; cold, numbness and tingling in limbs; dizziness; dyspnea; chest pain; and sore or dry throat. Furthermore, the country of residence was significantly associated (*p* < 0.01) with the frequencies of all post-vaccination side effects, except bleeding gums (*p* > 0.01). Unsurprisingly, the type of COVID-19 vaccine is significantly associated with all the frequencies of all post-vaccination side effects, except swollen ankles and feet, bleeding gums, and nosebleeds. However, the number of doses were only significantly associated with the following post-vaccination side effects: tiredness; fever; headache; injection site pain and swelling; joint pain; myalgia; nosebleed; chills; sleepiness and laziness, as well as the overall severity of side effects.

Moreover, the health status of participants (suffering from chronic diseases) is significantly associated with the frequencies of all post-vaccination side effects, except fever and vomiting, and the overall severity. Based on smoking status, there were statistical associations only with the frequencies of injection site pain and swelling, and sweating for no reason, and in the severity of post-vaccination side effects. There were significant associations (*p* < 0.01) between participants who suffered from food and/or drug allergies with the frequencies of all post-vaccination side effects except diarrhea and nosebleeds, and the overall severity. Interestingly, there were significant associations between experiencing COVID-19 vaccine hesitancy and related fears before vaccination, and the frequencies of all the post-vaccination side effects, and the overall severity. Experiencing COVID-19 infection before vaccination was significantly associated with all post-vaccination side effects, except swollen ankles and feet, vomiting, bleeding gums, nosebleeds and cough, as well as overall severity. The full results of χ^2^ tests and frequencies are shown in Table 4 and Table S1, respectively.

Moreover, according to χ^2^ tests, there was a significant association (*p* < 0.01) between experiencing COVID-19 vaccine breakthrough infection and vaccine type, but not number of doses received (Table 5). A correlation test showed significant correlations (*r* value > 40) between some countries (Algeria, Qatar, and Libya) and specific types of COVID-19 vaccines (SinoVac, Moderna, and Sputnik V, respectively) (Table S2).

### 3.7. Prediction of Post-Vaccination Side Effects Based on Predisposing Factors

Accuracy and Cohen’s kappa (κ) values were used to evaluate the prediction of post-vaccination side effects and overall severity using various ML tools. The best-predicted (Cohen’s κ > 20) side effects were tiredness, fever, injection site pain and swelling, headache, myalgia, joint pain, numbness and tingling in limbs, and sleepiness and laziness (Table 6). Moreover, based on Cohen’s κ values, GB was selected as the best predicting ML tool for further analysis. The feature importance for predisposing factors among the best predicted side effects was determined using GB; the global feature importance was determined according to interpretable global surrogate random forest (SRF) models. The results are shown in Table 7.

Subsequently, backward feature elimination from the least to the most important was combined with GB for selecting the most important input features for each of the investigated side effect based on jumps in Cohen’s κ values. For each side effect, the features with large drops in Cohen’s κ value (more than 2) were selected. Backward feature elimination findings for the best predicted symptoms using GB are shown in Table S3.

As shown in Table 8, vaccine type, gender, experiencing COVID-19 vaccine hesitancy and related fears before vaccination, and number of doses play significant roles in predicting the majority of the reported post-vaccination adverse effects. Based on the generalized linear models (GLM) or SRF scores, AstraZeneca and Moderna vaccines were the top contributing vaccines. Females, receiving two doses of a COVID-19 vaccine, and experiencing COVID-19 vaccine hesitancy and related fears before vaccination (in contrast with males, receiving one dose, and having no COVID-19 vaccine hesitancy or related fears) were more likely to predict the reported adverse effects. Being female appears to make one more likely for symptoms of tiredness and pain at the injection site than other factors, which is reasonable. Clearly, ML tools (GB in this case) can be used to predict some post-vaccination adverse effects (i.e., tiredness, fever, headache, paint at injection site, muscle pain, and feeling sleepy) based on a small number of predisposing factors such as vaccine type, gender, psychological fears, and number of doses, with a reasonable level of accuracy and Cohen’s κ values. Hot encoding was used with the selected predisposing factors (features) for each of the best predicting symptoms, and then global feature importance for composing the categories of each feature was determined according to interpretable global SRF models or generalized linear models (GLM). GB was used as AutoML, and the results are shown in Table 8.

## 4. Discussion

The present study is considered to be the first large-scale online post-COVID-19 vaccination survey of Arab populations, as well as their perceptions towards COVID-19 vaccines. A wide range of side effects was assessed and the most reported were tiredness, injection site pain and swelling, sleepiness and laziness, headache, myalgia, fever, joint pain, dizziness, chills, anxiety and sleep disorders, and numbness and tingling in limbs. Although these side effects are non-life-threatening, 9% of the participants experienced severe side effects. A few studies have assessed the potential side effects of COVID-19 vaccines in Arab countries, and none of them were multinational studies (Table 9). These studies also confirmed that the abovementioned side effects are the most redundant following COVID-19 vaccination.

Similar to the findings of previous studies (Table 9), participants experienced more side effects after the administration of the AstraZeneca vaccine, followed by the Pfizer-BioNTech vaccine and then Sinopharm vaccine. In total, the highest proportion of participants enrolled in these studies was those who were vaccinated with the AstraZeneca and Pfizer-BioNTech vaccines, while a smaller proportion received the Sinopharm vaccine. The present study involved a good number of participants who were vaccinated with Sputnik V (*n* = 587) and SinoVac (*n* = 468) vaccines. Furthermore, none of the previous studies were from the Arab countries in Africa, while the present study included six Arab nations in Africa (i.e., Egypt, Algeria, Tunisia, Libya, Morocco, Sudan, and Mauritania).

This study showed that, compared to their male peers, females were more likely to suffer from post-vaccination side effects, except bleeding gums and nosebleeds, and they also experienced these side effects at higher severity levels. Due to differences in hormonal homeostasis and genetic makeup, males and females tend to react differently to COVID-19 vaccines. This is not surprising, since it was well-known, even before the COVID-19 pandemic, that biological sex differences could influence the vaccine uptake, responses, and outcome [89]. Recent studies showed that the side effects of the Pfizer-BioNTech [87,90,91], AstraZeneca [90,91,92], Sinopharm [12], Sputnik V [11], SinoVac [91,93], Johnson & Johnson and Moderna [90] vaccines were significantly more frequent in females. With a view to reducing post-vaccination side effects in females and increasing immunogenicity in males, Ciarambino et al. [94] recommended that the vaccine development should be sex-specific, and that sex-related variables should be examined in pre-clinical and clinical vaccine trials. This should help in promoting the successful prevention of a COVID-19 pandemic by mass vaccination. Moreover, the comparison of age groups showed that participants aged 20 to 39 years were more likely to experience almost the majority of post-vaccination side effects, and they constituted the largest proportion of participants who suffered from severe side effects. Studies on different populations also showed that the side effects of different COVID-19 vaccines were significantly more frequent in younger individuals compared to in the elderly [11,90,91,92].

Compared to general populations, healthcare workers were less likely to experience the following side effects: haziness or lack-of-clarity in eyesight; swollen ankles and feet; abdominal pain; diarrhea; itchy or irritated skin and allergic reactions; sweating for no reason; cold, numbness and tingling in limbs; dizziness; dyspnea; chest pain; and sore or dry throat. This could be attributed to the positive attitude of healthcare workers toward COVID-19 vaccination [95]. However, they were more likely to suffer from fever. Furthermore, the frequencies of several post-vaccination side effects were significantly different based on the country of residence. An example is that, although the largest proportion of participants was from Lebanon, they were among the smallest proportions for all post-vaccination side effects, and the majority of them experienced mild side effects, or even no side effects, 45% and 36%, respectively. The previous example is by no means unique. Although these differences still need further large sample size observational studies, the current limited evidence with the past experiences among other types of viral vaccines indicates that adverse effects might be attributed to several factors, including ethnicity, lifestyle, and knowledge and attitude towards COVID-19 vaccination, and their related factors, such as trust in the accuracy of the measures taken by the government, education, and history of recommendation [95,96,97]. Although the Pfizer-BioNTech is the most administered vaccine in the Arab world, the frequencies of some types of COVID-19 vaccines were differed based on the country. Correlation test showed significant correlations between Algeria and the SinoVac vaccine, Qatar and the Moderna vaccine, and Libya and the Sputnik V vaccine (Table S2; *r* = 57, 64, and 42, respectively). This may indicate that these vaccines were most commonly administered in the correlated countries.

On the other hand, although all COVID-19 vaccines mostly cause similar post-vaccination side effects, the frequency and severity of these side effects were significantly associated with vaccine type. Generally, both Johnson & Johnson and AstraZeneca vaccines were associated with more side effects at moderate to severe levels, followed by the Moderna, Pfizer-BioNTech, Sputnik V, Sinopharm, and SinoVac vaccines. Although this is the first study comparing the possible side effects of all of these vaccines, the results were relatively consistent with the findings of previous studies [13,78,80,88]. Specifically, frequencies of post-vaccination side effects, except swollen ankles and feet, bleeding gums and nosebleeds, varied based on the type of COVID-19 vaccine. Notably, the participants who received the AstraZeneca vaccine were more susceptible to experience the rest of the post-vaccination side effects. However, despite of the vaccine type, there was a significant association between receiving the second dose and experiencing fever, headache, injection site pain and swelling, joint pain, myalgia, nosebleeds, chills, sleepiness and laziness. A study by Andrzejczak-Grządko reported that the majority of individuals with Pfizer-BioNTech vaccine experienced more side effects after the second dose than the first dose [98]. Moreover, in the present study, the majority of participants who suffered from moderate to severe side effects were vaccinated with the second dose, while they counted as the smallest proportion of participants who experienced mild or even no side effects. These findings were in line with the announcement of the Centers for Disease Control and Prevention (CDC), which stated that side effects possibly present after the second dose of a COVID-19 vaccine may be more intense [99].

Although suffering from chronic diseases was significantly associated with the frequencies and severity of post-vaccination side effects (Table 4), there were no differences between them in terms of frequencies and the severity of post-vaccination side effects. Participants who had more than one chronic disease were more susceptible to experience post-vaccination side effects, except fever and vomiting. Moreover, those participants were more likely to experience post-vaccination side effects with moderate to severe levels. These findings support the results of a study from Saudi Arabia by Alghamdi et al., which showed that the presence of chronic diseases correlated with the development of post-vaccination side effects [84]. Moreover, smokers were more susceptible to experience sweating for no reason, whereas non-smokers were more susceptible to experience injection site pain and swelling. The influence of smoking on immunological responses to viral vaccines have been assessed as early as the 1990s. Winter et al. studied the serological responses to hepatitis B vaccine at regular intervals among healthcare workers, and they reported that smoking had a significant adverse effect on their antibody responses [100]. In contrast, a study by Cruijff et al. showed that the efficacy of influenza vaccination was greater in smokers than in non-smokers [101]. These findings indicated that smoking may influence immunologic responses to COVID-19 vaccines. Hence, such a crucial hypothesis needs to be investigated in future studies, especially since no studies have covered it yet.

Participants with food and/or drug allergies were more susceptible to experiencing post-vaccination side effects, except diarrhea and nosebleeds. Moreover, they were at higher risk of developing moderate to severe side effects. In a recent study, 429 individuals with known history of allergic reactions (aeroallergens or insect bite, food, latex, or contrast media or prior non-anaphylactic reaction to a single drug group or those who had chronic urticarial) received the Pfizer-BioNTech vaccine, and allergic reactions were recorded. After the first dose, 420 patients (97.9%) had no immediate allergic reactions, 6 (1.4%) developed mild allergic events, and 3 (0.7%) had anaphylactic reactions. Among 218 patients who received the second dose of Pfizer-BioNTech vaccine, 214 (98.2%) had no allergic reactions, and 4 patients (1.8%) had mild allergic reactions [102]. In a meta-analysis of 14 studies, receiving the Pfizer-BioNTech vaccine was significantly associated with higher anaphylactic reactions and lower non-anaphylactic reactions compared to the Moderna vaccine [103].

There was a significant association between previous COVID-19 infection (before vaccination) and experiencing post-vaccination side effects. This result was consistent with the findings of a study from Italy by Ossato et al. [104]. Except for swollen ankles and feet, vomiting, bleeding gums, nosebleeds and cough, participants who experienced pre-vaccination COVID-19 infection were more susceptible to experiencing the rest of the post-vaccination side effects.

Interestingly, following COVID-19 vaccination, a total of 29 participants stated that they were diagnosed by a doctor with thrombocytopenia, and 22 participants experienced thrombosis, while 10 participants were diagnosed with both thrombocytopenia and thrombosis. Not surprisingly, those participants who suffered from thrombosis were vaccinated with the AstraZeneca, Pfizer-BioNTech, and Johnson & Johnson vaccines, n = 13, 8, and 1, respectively (Table 10). Despite being extremely rare, COVID-19 vaccine-induced thrombosis cases were mostly reported among individuals who had received the AstraZeneca vaccine, and less commonly after the Pfizer-BioNTech and Johnson & Johnson vaccines [13,105,106,107]. Interestingly, although the largest proportion of participants in this study was from Lebanon, where the predominant vaccine was Pfizer-BioNTech (51.5%), none of them experienced thrombosis. This variation between populations might be attributed to lifestyle and genetic susceptibility factors [108].

In the earliest studies on the safety of different types of COVID-19 vaccines, which comprised tens of thousands of individuals, no significant safety concerns were recorded, and the potential for serious health consequences (such as thrombocytopenia and thrombosis) has remained astonishingly low following the vaccination of more than 400 million individuals globally to date. It is not unexpected, therefore, that as more individuals are vaccinated and the follow up is extended, new reports of vaccination side effects would emerge [109].

For instance, there have been reports of immune thrombocytopenia and hemorrhage without thrombosis, following the administration of the messenger RNA (mRNA)-based vaccines manufactured by Moderna and Pfizer–BioNTech [107]. According to a case series by Hippisley et al., following the initial dose of the AstraZeneca vaccine, there was a higher risk of thrombocytopenia, venous thromboembolism, and other infrequent arterial thrombotic occurrences, in comparison to the first dose of the Pfizer-BioNTech vaccine, which showed an elevated incidence of arterial thromboembolism and ischemic stroke [106]. After the first injection of both vaccines, an elevated risk of cerebral venous sinus thrombosis was discovered a week later after receiving the Pfizer-BioNTech vaccine, compared to the AstraZeneca vaccine. In addition, according to our previous study, the majority of the individuals who consulted a doctor or were hospitalized had mild side effects. However, during the first 24 h after receiving the second dose of either Pfizer-BioNTech or AstraZeneca vaccines, six vaccinated individuals were diagnosed with thrombocytopenia, and two were also diagnosed with thrombosis [13]. In another study, the authors described the adverse effects of post-COVID-19 vaccination reported from 14 cases in a major hospital in Saudi Arabia. Among five serious cases, cerebral venous thrombosis (CVT) was reported in two cases 14 days after administering the AstraZeneca vaccine [110].

Similarly, Schultz et al. reported that, seven to ten days after administering the first dose of AstraZeneca vaccine, five individuals developed venous thrombosis and thrombocytopenia. The individuals were healthcare workers aged between 32 and 54 years, and all of them showed the significant production of antibodies to platelet factor 4 (PF4) and polyanions (P) complex (anti–PF4/P antibodies) [111]. It is believed that the five cases in the above study constitute an infrequent vaccine-related variation of spontaneously heparin-induced thrombocytopenia, called vaccine-induced immune thrombotic thrombocytopenia, since they occurred in a community of more than 130,000 immunized people. Furthermore, a recent review analyzed the case reports of 40 patients who suffered from vaccine-induced thrombotic thrombocytopenia after receiving adenoviral vector vaccines, Johnson & Johnson (*n* = 12) and AstraZeneca (*n* = 28) [105]. The comparison between the two vaccines showed similar symptoms and mortality, while in cases with the AstraZeneca vaccination, the CVT presented earlier with less thrombosis and intracerebral hemorrhage, and higher D-dimer and activated partial thromboplastin time (aPTT) levels. Furthermore, almost all patients were positive for anti-PF4/heparin antibodies and heparin-induced thrombocytopenia (HIT) antibodies, despite the type of vaccine received [105]. A case series, from Germany and Austria, included 9 CVT and three splanchnic vein thrombosis and other thrombosis cases after AstraZeneca vaccination. A total of five patients had disseminated intravascular coagulation, while six patients died. In the presence of PF4 independent of heparin, all patients who tested positive for anti-PF4/heparin antibodies were also positive on the platelet-activation assay. Furthermore, platelet activation was inhibited by high levels of heparin, Fc receptor–blocking monoclonal antibody, and immune globulin. This report showed an association between AstraZeneca vaccination and the rare development of immune thrombotic thrombocytopenia mediated by platelet-activating anti-PF4 antibodies, which clinically mimics autoimmune HIT [112].

Worries regarding the same risks have recently surfaced among a few persons who received the Pfizer-BioNTech vaccine. Notwithstanding, all blood tests (particularly platelet count and clotting-related assays) being normal, a 66-year-old female was identified with deep vein thrombosis, according to a report from Italy [113]. In a similar cohort, the chances of these events following vaccination were substantially lower than those linked to SARS-CoV-2 disease [108]. Therefore, following the initial doses of the AstraZeneca and Pfizer-BioNTech vaccines, elevated risks of hematologic and circulatory incidents that resulted in hospitalization or death were seen at brief periods. The chances of most of these outcomes were much greater and lasted longer after SARS-CoV-2 exposure than after vaccination [13]. Obviously, the benefits of obtaining a COVID-19 vaccine still outweigh the risks; the mortality [114], thrombocytopenia and thrombosis [115,116] risks of COVID-19 are still much greater.

The participants were asked whether they experienced other side effects that were not mentioned above. The most redundant side effects were lower back pain, menstrual dysfunctions, and erectile dysfunction and loss of libido (sex drive), *n* = 54, 39, and 12, respectively. Although the current evidence of COVID-19 vaccines’ effect on fertility is very limited, and several fertility societies have excluded this possible effect, it remains one of the reasons for vaccine hesitancy, especially among pregnant women or those who are trying to get pregnant [117]. Recently, undocumented reports have been raised about the potential adverse effects of COVID-19 vaccines on the menstrual cycle. The National Institutes of Health (NIH) endorsed that COVID-19 vaccines may affect the menstrual cycle, and observational studies are required to understand the exact mechanisms of action and to identify those women who are more likely to be affected. In addition to psychological aspects, menstrual dysfunctions following the COVID-19 vaccination could be attributed to the inflammatory mediators that the human body produces in response to receiving a vaccine. These mediators (i.e., cytokines and chemokines) potentially enter the uterus and stimulate the immune cells, which might cause abnormal menstruation timing or increase the release of prostaglandins that can increase the pain or other symptoms [118,119]. Furthermore, studies to assess rare cases of erectile dysfunction and loss of libido after administration with a COVID-19 vaccine are still scarce. Therefore, future studies should investigate these interesting side effects, in order to understand the physiological mechanisms that underlie each of them.

The COVID-19 outbreak prompted the creation of extremely potent immunizations that were manufactured at an extraordinary pace using a variety of vaccine development platforms. This rapidly achieved process was surrounded by many rumors and conspiracy theories, which consequently resulted in high rates of vaccine hesitancy. There are many reports, from almost all Arab countries, confirming that the fear of serious post-vaccination side effects and complications is the main reason behind the high rates of vaccine hesitancy [18,19,21,22,23,24,25,26,27,28,29,30,31,32,33,34]. In this study, a high rate of vaccine hesitancy (51%) was reported among the participants before receiving a vaccine. In Arab countries, vaccine rollout faces many challenges, such as limited supplies, difficulties in proper transport and delivery of vaccines, and in ensuring that adequately trained personnel are available for vaccine administration. However, one of the other major barriers to delivering adequate vaccines is the rate of vaccine hesitancy [17]. Vaccine hesitancy is a concern with all types of vaccination in general [120], but peaked in the COVID-19 pandemic. Studies from different regions of the world found that COVID-19 vaccine hesitancy varied significantly [121]. Differences in acceptance rates among 19 countries included in the Lazarus et al. study ranged from almost 90% (in China) to less than 55% (in Russia) [121]. Factors related to COVID-19 vaccine hesitancy, as reported in the literature, included general personal beliefs about vaccines, safety concerns, inadequate information about vaccines, their risk–benefit ratio, the role of natural immunity, and mistrust in the pharmaceutical industry, healthcare professionals and governments [122,123,124]. In the present study, vaccine hesitancy and related fears before vaccination were significantly associated with experiencing all post-vaccination side effects, and suffering from moderate to severe side effects. Interestingly, participants who did not experience vaccine hesitancy and related fears before vaccination were less likely to experience side effects after vaccination.

The present study reported that a total of 471 (4.7%) participants experienced COVID-19 vaccine breakthrough infection. Experiencing COVID-19 vaccine breakthrough infection were significantly associated with vaccine type (*p* < 0.01), whereas it was not associated with number of doses. Almost 8% of participants who received the AstraZeneca vaccine experienced COVID-19 vaccine breakthrough infection, which is the largest proportion compared to the most commonly reported vaccines in this study, Pfizer-BioNTech and Sinopharm vaccines (3% and 6% respectively). According to CDC data, as of 30 April, 2021, among 101 million people in the United States who had been fully vaccinated against COVID-19, 10,262 (0.01%) experienced vaccine breakthrough infections [125]. Most of these cases were females (*n* = 6446; 63%), with an interquartile age range of 40 to 74 years. Generally, most vaccine breakthrough infections were asymptomatic or mild. Only 995 people were known to be hospitalized, of which 289 were asymptomatic or admitted for reasons unrelated to their COVID-19 diagnosis. A total of 39 (2.6%) vaccine breakthrough infections were reported in a study that involved 1497 healthcare workers who had been fully vaccinated with Pfizer-BioNTech vaccine, and they were symptomatic or had known infection exposure [126]. Interestingly, two-thirds of breakthrough infection cases had mild symptoms and none required hospitalization, while the rest were asymptomatic. The study found a significant correlation between the occurrence of breakthrough infections and neutralizing antibody titers within the week before the molecular diagnosis [126]. However, higher rates of breakthrough infections (4.7%) were reported in the present study. Although some reports have shown that certain variants of concern were more prevalent in individuals with COVID-19 vaccine breakthrough infection [127,128,129], further epidemiological studies are required to confirm the presence of immunity-evading variants, especially with the emerging reports, which have shown a similar proportion of these variants among vaccine breakthrough infection cases and the general population [126,130,131].

On the basis of input descriptors (i.e., gender, age, education level, being a healthcare worker, country, suffering from chronic diseases, being a smoker, suffering from food and/or drug allergies, experiencing COVID-19 infection before receiving any vaccine dose, experiencing COVID-19 vaccine hesitancy and related fears before vaccination, type of COVID-19 vaccine, interval between receiving a COVID-19 vaccine and participating in this study, number of doses, experiencing COVID-19 vaccine breakthrough infection, time of breakthrough infection), ML tools (i.e., XGBoost, RF, MLP, PNN, LibSVM (nu), LibSVM (C), AdaBoost, GB, KNN, K*, and LWL) were used to predict different post-vaccination side effects. Table 6 shows that learners attained varying levels of accuracy, prompting us to utilize Cohen’s κ value as an additional success criterion for the ML models that resulted. Cohen’s κ value is a more robust metric, because it accounts for the potential of chance prediction. Cohen’s κ values of 0 to 0.20 are regarded as minor, 0.21 to 0.40 are considered fair, 0.41 to 0.60 are considered moderate, 0.61 to 0.80 are considered substantial, and 0.81 to 1.00 are considered to be in almost perfect agreement [132].

The algorithms RF, XGboost, MLP, GB, Adaboost, and K* produced good accuracy and reasonable Cohen’s κ values. KNN, LWL, LibSVM (c), and LibSVM (nu) had lower accuracy and Cohen’s κ values, whereas PNN was the least accurate ML tool and had the lowest Cohen’s κ value in our case. PNN is an implementation of a statistical procedure known as kernel discriminant analysis [133]. It has a number of drawbacks, including delayed network execution due to several layers and high memory needs, among others. In our research, the best forecasting ML tools were GB and RF. GB classifiers are a set of machine learning algorithms that integrate a number of weak learning models to form a powerful predictive model. When doing GB, decision trees are commonly employed. GB models are gaining popularity as a result of their ability to classify complex datasets [134].

For the reasons stated above, GB was chosen as AutoML in the global feature importance, and the top predicted post-vaccination side effects (i.e., tiredness, fever, headache, injection site pain and swelling, myalgia, limb numbness and tingling, and sleepiness and laziness) were chosen for further investigation. For the classification of each of these side effects, SRF was employed to examine global feature importance (as shown in Table 7). The goal of this stage is to rank the factors that predispose one to each post-vaccination side effect, from the least to the most important. Being a healthcare worker, for example, was determined to be a non-significant predisposing factor for predicting fever, whereas the type of vaccine was determined to be the most important factor. Table 7 shows that the type of vaccine was one of the most important factors for all of the side effects studied.

A backward elimination strategy was used to reduce the number of predisposing input factors used to forecast each of the analyzed adverse effects. Factors were deleted one by one, starting with the least important one, and accuracy and Cohen’s κ values were checked after each removal phase (Table S3). Asterisks identified and annotated features that had high jumps in Cohen’s κ values (>2), and the minimum number of selected features, were utilized to determine the global feature importance. For example, number of doses, experiencing COVID-19 vaccine hesitancy and related fears before vaccination, gender, type of COVID-19 vaccine, and country were chosen as the main predisposing input factors to predict sleepiness.

After that, distinct components of the main predisposing factors were hot encoded, global feature importance was calculated using GB as AutoML, and SRF and GLM were utilized as weighting methods to calculate the importance of each component. Table 8 shows that vaccine type, gender, number of doses, and experiencing COVID-19 vaccine hesitancy and related fears before vaccination all play a role in predicting the majority of the reported post-vaccination adverse effects. Both AstraZeneca and Moderna are the top contributing vaccines (based on GLM or SRF scores). Females, receiving two doses, and with fears (in contrary to males, receiving one dose, and having no fears) have more weights in predicting the reported adverse effects (as shown in Table 8). Being a female appears to carry greater weight in terms of tiredness and pain at the injection site than other factors. Clearly, machine learning (GB in this case) can be used to predict some post-vaccination adverse effects (i.e., tiredness, fever, headache, paint at injection site, muscle pain, and feeling sleepy), based on a small number of predisposing factors, such as vaccine type, gender, psychological fears, and number of doses, with a reasonable level of accuracy and Cohen’s κ values. To the best of our knowledge, this is the first study to apply extensive ML algorithms to predict various post-vaccination side effects using demographic and patient data as input features, and to weight the importance of different features in the prediction process.

In recent years, artificial intelligence (AI), and in particular ML, have expanded significantly in the context of data analysis and computing, allowing applications to perform intelligently. ML is one of the most popular current technologies in the fourth industrial revolution, since it allows systems to learn and improve from experience without having to be explicitly coded [135]. In the pandemic era, ML and deep learning (DL) offer a simple way of rapid COVID-19 screening and recognize possible high-risk patients, thereby maximizing care services and preventing serious symptoms [136]. The COVID-19 investigation made substantial use of ML and AI. A total of 130 publications were involved in a systematic review by Syeda et al. [137], while computational epidemiology, early detection and diagnosis, and disease progression were the three topics identified based on AI applications used to tackle the COVID-19 crisis. The computational epidemiology theme defined 71 (54.6%) of the 130 publications as focusing on predicting the COVID-19 outbreak, the influence of containment policies, and prospective drug discoveries. The early detection and diagnosis topic was then assigned to 40 of 130 (30.8%) publications that used AI approaches to detect COVID-19, utilizing patients’ radiological images or laboratory test results [137]. In a study by Shahid et al., the authors took a look at how ML has helped to combat the virus thus far, focusing on screening, forecasting, and vaccine development. They offered a complete overview of the ML algorithms and models that can be utilized on this mission to help in combating the pandemic [138].

Among the popular ML tools that were utilized in the fight against COVID-19, Gutierrez et al. used GB decision trees to estimate the risk of hospitalization within 30 days of a SARS-CoV-2 infection diagnosis, and Shapley values were used to assess variable relevance [139]. They employed the XGBoost technique to create a GB model, and compared its performance to four empirical risk stratification factors based on age and the number of comorbidities. Using routinely collected health administrative data, they constructed and verified an accurate risk stratification model [140]. The authors reported that risk stratification based on routinely gathered health data could help with COVID-19 management at the population level [139]. Furthermore, GB was used in modeling the impact of temperature and humidity on the transmission rate of COVID-19 in India [141]. Kaliappan et al. compared the performance of multiple non-linear regression techniques for COVID-19 reproduction rate prediction, including support vector regression (SVR), KNN, RF, GB, and XGBoost, as well as the impact of feature selection algorithms and hyperparameter tuning [140]. For the COVID-19 reproduction rate prediction, sixteen features (for example, total cases per million and total deaths per million) related to significant parameters such as testing, death, positive rate, active cases, stringency index, and population density were taken into account. The performances of algorithms with and without feature selection were similar, but a remarkable difference was seen with hyperparameter tuning [140].

Moreover, the ability to predict the severity of COVID-19 will considerably enhance care delivery and resource allocation, lowering mortality risks, particularly in developing countries. Many patient-related factors, such as pre-existing comorbidities, influence illness severity, and can be utilized to help predict disease severity. It was shown that several clinical parameters quantifiable in blood samples may distinguish between healthy persons and COVID-19-positive patients, and it demonstrated the utility of these parameters in predicting the severity of COVID-19 symptoms in the future [142]. Furthermore, MLP, XGBoost, RF, and K* were utilized to predict the severity of post-vaccination side effects among COVID-19 vaccine recipients in Jordan [13]. The RF, XGBoost, and MLP all had high accuracies (0.80, 0.79, and 0.70, respectively) and Cohen’s kappa values (0.71, 0.70, and 0.56, respectively), based on the type of vaccine, demographic data, and side effects. The study showed that, based on the input data, ML can also be used to forecast the severity of side effects, and thus projected severe cases may require additional medical attention, or possibly hospitalization [13].

The current rapid and exponential increase in the number of patients has prompted the use of AI approaches to predict the likely outcomes of an infected patient, in order to provide suitable therapy. Iwendi et al. developed a fine-tuned RF model with the AdaBoost algorithm as a boosting technique [143]. The COVID-19 patient’s geographic area, travel, health, and demographic data were used in the model to estimate the severity of the illness and the likelihood of recovery or death. On the dataset used, the model has an F1 score of 0.86 and an accuracy of 94%. The data analysis demonstrated a correlation between patient gender and death, as well as the fact that the majority of patients were aged 20 to 70 years old [143]. A clustered random forest technique was developed in another study to predict COVID-19 patient mortality [144]. By reviewing the demographic data for COVID-19 patients, they were able to uncover the underlying variability of patient frailty. They discovered that their clustered RF method outperforms other published methods in terms of prediction. They also discovered that a follow-up analysis using neural network modeling and k-means clustering can reveal the type and magnitude of COVID-19-related mortality risks [144].

In a study by Sharma et al., SVM was am ML classifier model utilized for disease classification (normal individuals vs. COVID-19 patients) [145]. By applying a modified cuckoo search algorithm and a hyperparameter optimization technique, the classifier’s classification accuracy can be improved. A hybrid feature selection technique as a minimum redundancy maximum relevance (mRMR) algorithm was used to select from high-dimensional data [145]. Furthermore, supervised ML algorithms were employed in a study by Ahamad et al. to identify the presentation features predicting COVID-19 disease diagnoses with high accuracy [146]. Features included age, gender, observation of fever, history of travel, and clinical details, such as the severity of cough and incidence of lung infection. Several machine learning algorithms were employed for the collected data and found that the XGBoost algorithm performed with the highest accuracy (>85%) to predict and select features that correctly indicate COVID-19 status for all age groups. Statistical analyses revealed that the most frequent and significant predictive symptoms are fever (41.1%), cough (30.3%), lung infection (13.1%), and runny nose (8.43%). Meanwhile, 54.4% of the people examined did not develop any symptoms that could be used for diagnosis [146].

In a recent study by Canas et al., ML was utilized to disentangle post-vaccination side effects from early COVID-19 infection [147]. The authors indicated that, although there were some differences in symptom prevalence and distribution between positive and negative individuals, these could not be used robustly to discriminate between groups, including using ML [147]. Another study aimed to discover possible common causes for post-vaccination side effects in order to predict them [47]. They looked at patient medical records as well as data on post-vaccination effects and outcomes. Different statistical methodologies were used to analyze the data, which were then followed by a set of ML classification algorithms. Similar characteristics were shown to be significantly associated with poor patient reactions in the majority of cases. Prior infections, hospitalization, and SARS-CoV-2 re-infection were among them. Patient age, gender, allergic history, taking other medications, type-2 diabetes, hypertension, and heart disease were the most significant pre-existing factors associated with a poor outcome and a long stay in the hospital [47]. Pyrexia, headache, dyspnea, chills, fatigue, various types of pain, and dizziness are the most significant clinical predictors, according to the findings. ML classifiers using medical history were also able to identify which patients were most likely to have a complication-free vaccination, with an accuracy rate of more than 85%. Through classification methodologies, their study reveals the profiles of individuals who may require further monitoring and care in order to reduce bad consequences. Allergy susceptibility and the incidence of heart disease or type-2 diabetes were important factors in achieving these reactions [47].

On the other hand, AutoML systems are data science assistants that scan data for novel features, pick appropriate supervised learning models, and optimize their parameters. The Tree-based Pipeline Optimization Tool (TPOT), using strongly typed genetic programming (GP) to provide an efficient analysis pipeline for the data scientist’s prediction issue, was created for this purpose [61]. In the realm of data mining, supervised ML algorithms have emerged as a popular strategy. The use of health data to predict disease has recently been identified as a possible application area for these technologies. Extensive research efforts were done to find studies that used more than one supervised ML algorithm to predict a particular disease. For distinct categories of search items, two databases (Scopus and PubMed) were searched [148]. As a result, a total of 48 articles for a comparison of supervised ML algorithms for disease prediction were selected. The SVM algorithm was found to be the most commonly used (in 29 research studies), followed by the Naive Bayes algorithm (in 23 research studies). In comparison, the RF algorithm showed greater accuracy. In nine of the 17 studies in which it was used, RF had the highest accuracy, at 53%. This was followed by SVM, which came out on top in 41% of the research examined [148].

## 5. Study Strengths and Limitations

In the current study, some limitations should be considered during the interpretation of the results. The questionnaire was distributed online using social media platforms, which may bias the participant’s proportions in different groups, such as age and socioeconomic demographics, with the ability to regularly access these platforms. For example, individuals aged 20 to 40 years are familiar with this technology, so we expected that large numbers of this group would participate, whereas there are lower proportions of people aged over 50 who use social medial, and fewer were expected to participate in this survey. Furthermore, it is difficult to reach individuals who have no internet connection (e.g., in remote areas). The distribution of the questionnaire via social media would increase the information bias due to differences resulting from exposure, interpretation, or the misclassification of side effects, and the variability in tolerance thresholds from patient to patient, since the side effects were not clinically confirmed by physicians. According to the inadequate resources and the time-sensitive environment of the pandemic, it was hard to include participants from all Arab countries, and the number of involved participants from a few countries was considered modest. However, a large number of participants covered most of the countries, which may have reduced the sampling bias. There were few participants who received some COVID-19 vaccines, such as Johnson & Johnson; therefore, we would not be able to accurately assess the side effects of these vaccines. Finally, close-ended answers (Yes/No) were used in the survey, while no open-ended responses were used, which limits the information provided by participants. Further studies are recommended to address post-vaccination side effects that would emerge after the third dose (complementary). The number of participants who had a drug or food allergy is small (1182/10,064); therefore, a large-scale study should assess post-vaccination side effects among people with a drug or food allergy.

In order to provide more reassurance regarding what people might expect following the administration of a COVID-19 vaccine, it would have been more efficient if this study determined humoral immunogenicity, along with the possible side effects of the COVID-19 vaccines. However, since it is a multinational study which involves the data of participants from different countries, it was difficult to collect blood samples, or even obtain their clinical data, to determine the humoral immunogenicity by measuring SARS-CoV-2 receptor-binding domain (RBD) antibody and the SARS-CoV-2 neutralizing antibody.

Despite these limitations, this study may still deliver necessary fundamentals and facts to health and governmental authorities to help in conducting effective vaccination campaigns in Arab communities that are still significantly affected with COVID-19, due to the hesitancy of their population toward receiving COVID-19 vaccines. Previous studies on the post-COVID-19 vaccinated side effect focused on a specific country or region, and to the best of our knowledge, this is the first large scale study comparing the post-vaccination side effects of different vaccines among the Arab world, involving more than 10,000 participants, which is a large sample size, and it allows one to generalize the results, at least among the Arab populations.

Moreover, the use of ML tools to predict the major common side effects is also considered one of the strengths of this study, which may enhance the novelty of the study and increase the validity and accuracy of the results. Furthermore, this is the first study to apply extensive ML algorithms to predict various post-vaccination side effects using demographic and patient data as input features, and to weight the importance of different features in the prediction process. Our unique methods may draw attention to the fact that only a few predisposing factors can be used to predict certain post-vaccination side effects.

Indeed, people with identifiable risk factors of experiencing the top predicted post-vaccination side effects (i.e., tiredness, fever, headache, injection site pain and swelling, myalgia, limb numbness and tingling, and sleepiness and laziness) might require additional strategies to strengthen their awareness and prevent severe side effects. For example, since the ML prediction showed that the type of COVID-19 vaccine is one of the most important predisposing factors for all the top predicted post-vaccination side effects, vaccine recipients should receive adequate awareness about the predicted side effects and the overall severity based on the type of vaccine they received. Vaccines recipients with predisposing factors of fever, for example, need to be aware of the importance of the continuous measuring of body temperature and the normal range, as well as when to use antipyretics and the recommended doses, and when they might need medical help and hospital treatment. With such information, these people can be well prepared for facing post-vaccination side effects, even if they face any of them more frequently or at higher severity levels compared to their peers. Therefore, they will be ready with the suitable measures to deal with the predicted side effects, which in turn, may assist in identifying avoidable hospital admissions, reducing the pressure on hospitals. This may also assist in building vaccine confidence among the population, encouraging more people to get vaccinated, ultimately leading to reduced risk of developing severe COVID-19 symptoms and serious complications, and fewer hospitalizations and deaths. Furthermore, this preparedness might help people, especially older adults and those with chronic diseases, to relieve the COVID-19 vaccine-related psychological stress which can influence the functioning of the immune system.

## 6. Conclusions

The Arab world is still grappling with the COVID-19 pandemic and its repercussions for public health. In addition to the known logistical challenges that are faced when rolling out mass vaccination campaigns in low- and middle-income countries, the present study reported a high rate of vaccine hesitancy among Arab populations. Although the authorized COVID-19 vaccines have proven to be effective and safe, similar to any therapeutics, they may cause a variety of side effects. These side effects are considered non-life-threatening, and the overall severity is mostly mild to moderate, while rare cases suffered from vaccine-induced immune thrombosis and thrombocytopenia. Most of these cases were vaccinated with the AstraZeneca vaccine, and less commonly with the Pfizer-BioNTech vaccine. Despite vaccine type and number of doses, rare cases of COVID-19 vaccine breakthrough infection were reported. Various predisposing factors (such as gender, age, smoking, country, type of COVID-19 vaccine, suffering from chronic diseases) were associated with the frequencies of post-vaccinations side effects and the overall severity. Furthermore, ML tools were used to predict the post-vaccination side effects based on predisposing factors, and the best forecasting tools were GB and RF. The global feature importance was calculated using GB (as AutoML), and both SRF and GLM were utilized as weighting methods to calculate the importance of each component. Vaccine type, gender, number of doses, and experiencing COVID-19 vaccine hesitancy and related fears before vaccination play a role in predicting the majority of the reported post-vaccination side effects. Certain predisposing factors have greater weight and importance as input data in predicting post-vaccination side effects. Based on the most significant input data, ML can also be used to predict these side effects; patients with certain predicted side effects may require additional medical attention or possibly hospitalization.

## Figures and Tables

**Table 1:** List of ML algorithms and evaluation tools that were used in the present study.

ML/Evaluation Tool	Principle	Settings	References
Random Forest (RF)	A multipurpose ML method for classification. RF is based on an ensemble of decision trees (DTs). Each tree predicts a classification independently and “votes” for the related class, and the majority of votes decide the overall RF predictions.	Splitting criterion is the information gain ratio; the number of trees is 100. No limitations were imposed on the number of levels or minimum node size. The accuracy was calculated using out-of-bag internal validation.	[49,50,51]
eXtreme Gradient Boosting (XGBoost)	XGBoost depends on the ensemble of weak DT-type models to create boosted, DT-type models. This system includes a new tree learning algorithm, a theoretically justified weighted quantile sketch procedure with parallel, and distributed computing.	Tree booster was implemented with depth wise grow policy, boosting rounds = 100, Eta = 0.3, Gamma = 0, maximum depth = 6, minimum child weight = 1, maximum delta step = 0, sub-sampling rate = 1, column sampling rate by tree = 1, column sampling rate by level = 1, lambda = 1, Alpha = 0, sketch epsilon = 0.03, scaled position weight = 1, maximum number of bins = 256, sample type (uniform), normalize type (tree), and dropout rate = 0.	[52,53,54]
Multilayer Perceptron (MLP)	An implementation of the RProp algorithm for multilayer feed forward networks. MLP has the capability to learn nonlinear models in real-time. MLP can have one or more nonlinear hidden layers between the input and output layers. For each hidden layer, diverse numbers of hidden neurons can be assigned. Each hidden neuron grants a weighted linear summation for the values from the previous layer, and the nonlinear activation function is followed. The output values were determined after the output layer transforms the values from the last hidden layer.	Maximum number of iterations = 100, number of hidden layers = 3, and number of hidden neurons per layer = 10.	[55,56]
K-Star (K*)	It is an instance-based classifier. The class of a test instance is dependent upon the class of those training instances similar to, as determined by some similarity function. It varies from other instance-based learners by using an entropy-based distance function.	Average column entropy curve is used for missing mode, and manual blend setting is 20%.	[57,58]
Accuracy	Evaluation of ML models	Accuracy = (TP + TN)/N TP is the true positive (correctly classified predictions), TN is true negative (truly classified predictions), and N is the total number of evaluated cases.	[37,59]
Cohen’s kappa (κ) value	Evaluation of ML models	Cohen’s κ = (P_0_ + P_e_)/(1 − P_e_) P_0_ is the relative observed agreement among raters (i.e., accuracy), and P_e_ is the hypothetical probability of chance agreement. This was carried out by using the observed data to calculate the probabilities of each observer randomly seeing each category. If the raters are in complete agreement, then Cohen’s κ = 1. If there is no agreement among the raters other than what would be expected by chance (as given by P_e_), Cohen’s κ = 0. Negative Cohen’s κ value implies the agreement is worse than random.	[59,60]
Compute Global Feature Importance	This application is a simple example of inspecting global feature importance for binary classification. In this example, the symptom data set is partitioned to training and test samples. Then, the black box model is trained on the pre-processed training data using the automated machine learning (AutoML) component. The Workflow Object capturing the pre-processing and the model is provided as an input for the global feature importance component together with the test data. The component provides the global feature importance according to interpretable global surrogate random forest models or generalized linear models (GLM).	AutoML: Models to train in AutoML = Gradient Boost, Metric for auto selection = Cohen’s κ value, hot encoding is used. Number of folds in cross validation = 4, Size of training set partition (%) = 80, Maximum amount of unique values in a categorical column = 100. Global feature Importance: Importance methods = Surrogate random forest and surrogate generalized linear model. Performance metric = Cohen’s κ value. The number of permutations = 3, Show top n features = 10, and maximum percentage of unique values in a categorical column = 100.	[61,62]
Probabilistic Neural Network (PNN)	A probabilistic neural network (PNN) is a type of feedforward neural network that is usually used to solve classification and pattern recognition tasks. A Parzen window and a non-parametric function are utilized to approximate the parent probability distribution function (PDF) of each class in the PNN method. The class probability of test data (new input data) is then estimated depending on the PDF of each class, and Bayes’ rule is used to allocate the class with the highest posterior probability to new input data. The risk of misclassification is reduced with this strategy.	PNN theta minus = 0.2 and theta plus = 0.4 and without specifying maximum number of epochs so that the PNN process is repeated until stable rule model is achieved.	[63,64]
Library for Support Vector Machines (LibSVM)	LIBSVM supports classification and regression by performing the sequential minimum optimization (SMO) algorithm for kernelized support vector machines (SVMs). SVM is an effective tool for both classification and regression. This operator supports the C-SVC and nu-SVC SVM types for classification tasks. The standard SVM uses a set of input data and predicts which of two potential classes the input belongs to for each given input, considering it a non-probabilistic binary linear classifier. An SVM training algorithm builds a model that allocates new examples to one of two categories based on a set of training examples that have been labeled as belonging to one of two categories. An SVM model is a representation of the examples as points in space, mapped so that the examples of the different categories are separated by a large distance. New examples are then mapped into the same space and classified according to which side of the gap they fall on.	C-SVM and nu-SVM. C methods were attempted, C and nu are regularization parameters that penalize misclassifications. C ranges from 0 to infinity while nu ranges between 0 and 1 and represents the lower and upper bound on the number of examples that are support vectors and that lie on the wrong side of the hyperplane. The following default settings were used in both SVM methods as implemented in the WEKA-KNIME (version 4.1.3) LibSVM node, these include: Kernel Cache (Cache Size = 40.0), kernel type is radial basis function: exp (−gamma×|u − v|^2^), and loss function is 0.1, kernel coefficients epsilon = 0.001 and Gamma = 0.00. However, in nu-SVM the optimized nu value of 0.1 was used (identified using Bayesian Optimization (TPE) implemented in KNIME).	[65,66,67,68]
Adaptive Boosting (AdaBoost)	AdaBoost algorithm is used as a statistical classification meta-algorithm. AdaBoost is adaptive in that it tweaks succeeding weak learners in favor of instances misclassified by earlier classifiers. It may be less likely to face the overfitting problem than other learning algorithms in particular situations. Individual learners may be poor, but as long as their performance is marginally better than random guessing, the final model will converge to a powerful learner.	Percentage of weight mass to base training on = 100, Random number seed = 1, Number of iterations = 10, and base is DecisionStump.	[69,70]
Gradient Boosting (GB)	GB is a machine learning technique that can be utilized for different applications, including regression and classification. It returns a prediction model in the form of an ensemble of weak prediction models, most commonly decision trees. The occurring approach is called GB trees when a decision tree is the weak learner; it usually outperforms random forest. A GB trees model is constructed in the same stage-wise manner as other boosting approaches, but it varies in that it allows optimization of any differentiable loss function.	Limit number of levels (tree depth) = 4, number of models = 10, and learning rate = 0.1	[71,72,73]
K-Nearest Neighbor (KNN)	KNN is either used for classification and regression, the input includes the k closest training examples in a data set. The output depends on whether KNN is employed for classification or regression. In classification, the output is a class membership. An object is classified by the overall vote of its neighbors, with the object being assigned to the class most common among its k nearest neighbors (k is a positive integer).	Number of neighbors to consider (k) = 5, weight neighbors by distance is on.	[74,75]
Locally Weighted Learning (LWL)	Locally Weighted Learning methods are non-parametric and the current prediction is done by local functions. The basic idea behind LWL is that instead of building a global model for the whole function space, for each point of interest a local model is created based on neighboring data of the query point.	The nearest neighbor search algorithm to use = LinearNNSearch, the number of neighbors used to set the kernel bandwidth = all, the weighting kernel shape to use = Linear, and base classifier is a Decision Stump.	[76]

**Table 2:** List of surveyed countries and demographic characteristics of the participants.

Country	Participants *n* (%)	Gender *n* (%)		Age (Year). *n* (%)				Education *n* (%)			Healthcare Worker *n* (%)	
		Male	Female	<20	20–39	40–59	>60	High School or Less	Undergraduate	Postgraduate	Yes	No
Lebanon	1946 (19)	843 (43)	1103 (57)	501 (26)	1156 (59)	247 (13)	42 (2)	187 (9)	1300 (67)	459 (24)	654 (34)	1292 (66)
Jordan	1714 (17)	523 (31)	1191 (69)	28 (2)	1211 (70)	442 (26)	33 (2)	179 (11)	1219 (71)	316 (18)	423 (25)	1291 (75)
Saudi Arabia	1561 (16)	665 (43)	896 (57)	32 (2)	825 (53)	576 (37)	128 (8)	211 (14)	980 (62)	370 (24)	537 (34)	1024 (66)
Iraq	934 (9)	501 (54)	433 (46)	48 (5)	633 (68)	233 (25)	20 (2)	111 (12)	704 (75)	119 (13)	252 (27)	682 (73)
Egypt	751 (7)	311 (41)	440 (59)	7 (1)	449 (60)	266 (35)	29 (4)	37 (5)	515 (69)	199 (26)	171 (23)	580 (77)
Palestine	531 (5)	187 (35)	344 (65)	37 (7)	360 (68)	117 (22)	17 (3)	31 (6)	419 (79)	81 (15)	250 (47)	281 (53)
Algeria	407 (4)	231 (57)	176 (43)	3 (1)	226 (55)	158 (39)	20 (5)	66 (16)	80 (20)	261 (64)	90 (22)	317 (78)
Tunisia	376 (4)	158 (42)	218 (58)	1	119 (32)	201 (53)	55 (15)	75 (20)	81 (22)	220 (58)	61 (16)	315 (84)
Syria	339 (3)	247 (73)	92 (27)	5 (1)	133 (39)	152 (45)	49 (15)	41 (12)	144 (43)	154 (45)	247 (73)	92 (27)
Libya	316 (3)	164 (52)	152 (48)	3 (1)	115 (36)	154 (49)	44 (14)	42 (13)	208 (66)	66 (21)	69 (22)	247 (78)
Qatar	263 (3)	160 (61)	103 (39)	1	142 (54)	111 (42)	9 (3)	14 (5)	188 (72)	61 (23)	63 (24)	200 (76)
Kuwait	239 (2)	126 (53)	113 (47)	0	121 (51)	113 (47)	5 (2)	36 (15)	167 (70)	36 (15)	36 (15)	203 (85)
Morocco	196 (2)	105 (54)	91 (46)	3 (2)	130 (66)	53 (27)	10 (5)	14 (7)	61 (31)	121 (62)	32 (16)	164 (84)
Bahrain	179 (2)	66 (37)	113 (63)	1	90 (50)	56 (31)	32 (18)	30 (17)	106 (59)	43 (24)	28 (16)	151 (84)
UAE	112 (1)	66 (59)	46 (41)	1	55 (49)	52 (46)	4 (4)	18 (16)	59 (53)	35 (31)	22 (20)	90 (80)
Oman	76 (1)	43 (57)	33 (43)	1 (1)	35 (46)	35 (46)	5 (7)	1 (1)	47 (62)	28 (37)	9 (12)	67 (88)
Sudan	63 (1)	33 (52)	30 (48)	0	50 (79)	12 (19)	1 (2)	10 (16)	32 (51)	21 (33)	14 (22)	49 (78)
Yemen	50	18 (36)	32 (64)	2 (4)	34 (68)	13 (26)	1 (2)	13 (26)	22 (44)	15 (30)	14 (28)	36 (72)
Mauritania	11	5 (45)	6 (55)	0	8 (73)	2 (18)	1 (9)	3 (27)	5 (45)	3 (27)	3 (27)	8 (73)
Total	10,064	4466 (44)	5598 (56)	674 (6)	5892 (59)	2992 (30)	505 (5)	1119 (11)	6337 (63)	2608 (26)	2975 (30)	7089 (70)

UAE, United Arab Emirates.

**Table 3:** Classification of participants based on types of COVID-19 vaccines and number of doses.

Vaccine	Participants *n* (%)	Dose *n* (%)	
		One	Two
Pfizer-BioNTech ^1^	5310 (52.8)	2948 (56)	2362 (44)
AstraZeneca ^2^	2087 (20.7)	1200 (57)	887 (43)
Sinopharm ^3^	1433 (14.2)	511 (36)	922 (64)
Sputnik V ^4^	587 (5.8)	299 (51)	288 (49)
SinoVac ^5^	468 (4.6)	306 (65)	162 (35)
Moderna ^6^	121 (1.2)	35 (29)	86 (71)
Johnson & Johnson ^7^	58 (0.6)	57 (98)	1 (2)
Total	10,064	5356 (53)	4708 (47)

Developer(s): ^1^ Pfizer Inc., New York City, NY, USA and BioNTech SE, Mainz, Germany; ^2^ AstraZeneca plc, Cambridge, UK; ^3^ China National Pharmaceutical Group Corporation, Beijing, China; ^4^ Gamaleya Research Institute of Epidemiology and Microbiology, Moscow, Russia; ^5^ Sinovac Biotech, Beijing, China; ^6^ Moderna Inc., Cambridge, MA, USA; ^7^ Janssen Vaccines, Leiden, The Netherlands.

**Table 4:** Assessment of the statistical association between predisposing factors and side effects of COVID-19 vaccines using χ^2^ tests.

Post-Vaccination Side Effects	Statistical Values	Predisposing Factors											
		Gender	Age	Education Level	Being a Healthcare Worker	Country	Type of COVID-19 Vaccine	Number of Doses	Suffering from Chronic Diseases	Being Smoker	Suffering from Food and/or Drug Allergies	Experiencing COVID-19 Vaccine Hesitancy and Related Fears before Vaccination	Experiencing COVID-19 Infection before Vaccination
Tiredness	χ^2^	348.81	216.92	50.86	3.48	470.66	381.94	23.71	48.16	5.88	10.41	214.27	59.66
	DF	1	3	2	1	18	6	1	10	1	1	1	1
	*p*	0.000	0.000	0.000	0.062	0.000	0.000	0.000	0.000	0.015	0.001	0.000	0.000
Anxiety, depression and sleep disorders	χ^2^	93.64	98.68	26.43	5.54	298.51	162.81	3.97	35.31	0.57	11.31	318.11	55.97
	DF	1	3	2	1	18	6	1	10	1	1	1	1
	*p*	0.000	0.000	0.000	0.019	0.000	0.000	0.046	0.000	0.452	0.001	0.000	0.000
Fever	χ^2^	66.59	114.72	15.32	9.05	492.59	706.12	57.02	21.30	3.72	11.58	84.99	13.84
	DF	1	3	2	1	18	6	1	10	1	1	1	1
	*p*	0.000	0.000	0.000	0.003	0.000	0.000	0.000	0.019	0.054	0.001	0.000	0.000
Headache	χ^2^	243.11	107.78	27.07	0.01	307.95	271.56	32.42	30.95	3.70	27.64	176.27	39.54
	DF	1	3	2	1	18	6	1	10	1	1	1	1
	*p*	0.000	0.000	0.000	0.910	0.000	0.000	0.000	0.001	0.054	0.000	0.000	0.000
Haziness or lack-of-clarity in eyesight	χ^2^	64.72	30.74	12.97	15.70	215.74	66.93	1.13	46.56	3.49	16.80	171.83	9.72
	DF	1	3	2	1	18	6	1	10	1	1	1	1
	*p*	0.000	0.000	0.002	0.000	0.000	0.000	0.287	0.000	0.062	0.000	0.000	0.002
Injection site pain and swelling	χ^2^	387.31	181.27	61.68	5.79	461.47	508.68	7.11	35.63	9.89	15.91	132.81	29.94
	DF	1	3	2	1	18	6	1	10	1	1	1	1
	*p*	0.000	0.000	0.000	0.016	0.000	0.000	0.008	0.000	0.002	0.000	0.000	0.000
Joint pain	χ^2^	187.85	122.97	22.08	4.04	389.40	327.40	42.95	63.59	0.09	16.66	189.04	38.26
	DF	1	3	2	1	18	6	1	10	1	1	1	1
	*p*	0.000	0.000	0.000	0.045	0.000	0.000	0.000	0.000	0.761	0.000	0.000	0.000
Swollen ankles and feet	χ^2^	117.35	22.95	7.01	18.60	120.82	7.89	1.43	124.63	0.71	10.89	112.60	6.18
	DF	1	3	2	1	18	6	1	10	1	1	1	1
	*p*	0.000	0.000	0.030	0.000	0.000	0.246	0.231	0.000	0.400	0.001	0.000	0.013
Myalgia	χ^2^	179.21	138.21	39.27	1.63	368.75	321.06	32.88	41.58	0.24	10.39	159.93	19.34
	DF	1	3	2	1	18	6	1	10	1	1	1	1
	*p*	0.000	0.000	0.000	0.201	0.000	0.000	0.000	0.000	0.627	0.001	0.000	0.000
Nausea	χ^2^	274.59	72.92	16.38	3.50	104.42	83.96	0.44	28.65	4.53	25.38	155.06	20.94
	DF	1	3	2	1	18	6	1	10	1	1	1	1
	*p*	0.000	0.000	0.000	0.061	0.000	0.000	0.507	0.001	0.033	0.000	0.000	0.000
Abdominal pain	χ^2^	134.48	72.89	9.14	14.43	151.04	44.69	4.51	34.72	0.08	13.60	142.77	33.27
	DF	1	3	2	1	18	6	1	10	1	1	1	1
	*p*	0.000	0.000	0.010	0.000	0.000	0.000	0.034	0.000	0.778	0.000	0.000	0.000
Diarrhea	χ^2^	33.21	43.37	4.60	17.71	143.62	22.58	2.51	30.88	0.05	5.38	78.07	39.61
	DF	1	3	2	1	18	6	1	10	1	1	1	1
	*p*	0.000	0.000	0.100	0.000	0.000	0.001	0.113	0.001	0.828	0.020	0.000	0.000
Vomiting	χ^2^	41.27	20.50	2.55	0.60	51.06	53.68	0.00	6.06	0.00	12.34	22.14	3.52
	DF	1	3	2	1	18	6	1	10	1	1	1	1
	*p*	0.000	0.000	0.279	0.438	0.000	0.000	0.966	0.810	0.972	0.000	0.000	0.061
Bruises on the body	χ^2^	90.06	20.69	5.77	2.05	101.13	33.58	0.01	24.71	0.03	12.77	35.97	15.82
	DF	1	3	2	1	18	6	1	10	1	1	1	1
	*p*	0.000	0.000	0.056	0.152	0.000	0.000	0.906	0.006	0.854	0.000	0.000	0.000
Bleeding gums	χ^2^	1.50	3.49	0.88	1.13	28.95	12.13	2.12	52.34	0.02	9.50	29.27	0.21
	DF	1	3	2	1	18	6	1	10	1	1	1	1
	*p*	0.220	0.322	0.643	0.288	0.049	0.059	0.146	0.000	0.876	0.002	0.000	0.650
Nosebleed	χ^2^	0.00	2.34	0.54	3.22	60.02	8.73	6.98	27.03	5.36	1.45	148.50	2.15
	DF	1	3	2	1	18	6	1	10	1	1	1	1
	*p*	0.983	0.504	0.763	0.073	0.000	0.190	0.008	0.003	0.021	0.229	0.000	0.143
Chills	χ^2^	158.18	102.65	16.33	0.97	433.68	454.30	77.70	37.08	0.04	13.95	87.51	11.70
	DF	1	3	2	1	18	6	1	10	1	1	1	1
	*p*	0.000	0.000	0.000	0.325	0.000	0.000	0.000	0.000	0.840	0.000	0.000	0.001
Itchy skin, or irritation and allergic reactions	χ2	58.98	12.02	0.60	12.36	92.68	18.55	0.18	52.73	0.81	59.50	81.86	9.47
	DF	1	3	2	1	18	6	1	10	1	1	1	1
	*p*	0.000	0.007	0.739	0.000	0.000	0.005	0.669	0.000	0.369	0.000	0.000	0.002
Sweating for no reason	χ^2^	37.25	28.36	8.14	10.11	155.91	177.94	0.85	45.68	14.80	21.38	144.52	39.40
	DF	1	3	2	1	18	6	1	10	1	1	1	1
	*p*	0.000	0.000	0.017	0.001	0.000	0.000	0.356	0.000	0.000	0.000	0.000	0.000
Cold, numbness and tingling in limbs	χ^2^	109.93	64.64	20.86	8.64	192.88	199.45	0.09	38.75	0.64	19.52	231.80	16.26
	DF	1	3	2	1	18	6	1	10	1	1	1	1
	*p*	0.000	0.000	0.000	0.003	0.000	0.000	0.758	0.000	0.424	0.000	0.000	0.000
Dizziness	χ^2^	285.06	94.20	25.85	11.04	335.10	145.06	1.50	27.40	5.69	16.35	56.85	29.22
	DF	1	3	2	1	18	6	1	10	1	1	1	1
	*p*	0.000	0.000	0.000	0.001	0.000	0.000	0.221	0.002	0.017	0.000	0.000	0.000
Clogged nose	χ^2^	32.47	37.68	11.81	1.52	148.96	31.09	0.00	35.31	1.00	19.87	40.92	18.33
	DF	1	3	2	1	18	6	1	10	1	1	1	1
	*p*	0.000	0.000	0.003	0.218	0.000	0.000	0.986	0.000	0.317	0.000	0.000	0.000
Runny nose	χ^2^	37.74	18.53	5.57	0.21	93.77	17.16	0.01	41.96	0.08	19.64	101.46	14.23
	DF	1	3	2	1	18	6	1	10	1	1	1	1
	*p*	0.000	0.000	0.062	0.645	0.000	0.009	0.940	0.000	0.774	0.000	0.000	0.000
Dyspnea	χ^2^	64.95	44.31	16.76	10.40	176.86	43.71	0.83	44.31	0.95	20.91	119.88	15.16
	DF	1	3	2	1	18	6	1	10	1	1	1	1
	*p*	0.000	0.000	0.000	0.001	0.000	0.000	0.363	0.000	0.329	0.000	0.000	0.000
Chest pain	χ^2^	33.30	57.66	13.37	12.47	246.59	59.47	0.28	53.52	2.46	22.91	208.47	24.24
	DF	1	3	2	1	18	6	1	10	1	1	1	1
	*p*	0.000	0.000	0.001	0.000	0.000	0.000	0.599	0.000	0.117	0.000	0.000	0.000
Sleepiness and laziness	χ^2^	284.72	173.50	38.46	4.84	309.15	135.99	7.45	34.98	1.01	11.35	204.67	35.19
	DF	1	3	2	1	18	6	1	10	1	1	1	1
	*p*	0.000	0.000	0.000	0.028	0.000	0.000	0.006	0.000	0.315	0.001	0.000	0.000
Irregular heartbeats	χ^2^	117.55	56.90	12.73	4.63	306.14	86.46	0.72	70.05	0.42	33.00	101.57	32.87
	DF	1	3	2	1	18	6	1	10	1	1	1	1
	*p*	0.000	0.000	0.002	0.031	0.000	0.000	0.396	0.000	0.515	0.000	0.000	0.000
Abnormal blood pressure	χ^2^	56.78	17.41	12.73	3.43	199.35	57.09	0.31	114.06	0.81	12.57	148.15	12.59
	DF	1	3	2	1	18	6	1	10	1	1	1	1
	*p*	0.000	0.001	0.002	0.064	0.000	0.000	0.579	0.000	0.367	0.000	0.000	0.000
Sore or dry throat	χ^2^	102.02	43.03	16.45	23.87	213.90	52.52	5.43	36.76	0.01	34.92	49.70	31.62
	DF	1	3	2	1	18	6	1	10	1	1	1	1
	*p*	0.000	0.000	0.000	0.000	0.000	0.000	0.020	0.000	0.917	0.000	0.000	0.000
Cough	χ^2^	22.65	27.59	14.40	2.63	112.21	21.98	0.57	41.05	0.15	15.67	227.95	4.36
	DF	1	3	2	1	18	6	1	10	1	1	3	1
	*p*	0.000	0.000	0.001	0.105	0.000	0.001	0.451	0.000	0.694	0.000	0.000	0.037
Severity of post-vaccination side effects	χ^2^	345.78	199.75	32.52	0.46	788.45	888.54	36.81	72.85	11.86	28.11	214.27	40.93
	DF	3	9	6	3	54	18	3	30	3	3	1	3
	*p*	0.000	0.000	0.000	0.928	0.000	0.000	0.000	0.000	0.008	0.000	0.000	0.000

χ^2^, chi-square; DF, degree of freedom; *p*, *p*-value (significant at ≤0.01).

**Table 5:** The χ^2^ testing of statistical associations of both vaccine type and number of doses with experiencing COVID-19 vaccine breakthrough infection.

Variable		COVID-19 Vaccine Breakthrough Infection	Real Value	Expected Value	DF	χ^2^	*p*
Vaccine type	AstraZeneca	No	1925	1988.56	6	76.98	0.000
		Yes	161	97.43			
	Pfizer-BioNTech	No	5141	5061.01			
		Yes	169	247.98			
	Sinopharm	No	1351	1366.06			
		Yes	82	66.93			
	Johnson & Johnson	No	54	55.29			
		Yes	4	2.70			
	Moderna	No	117	115.34			
		Yes	4	5.65			
	Sputnik V	No	563	559.58			
		Yes	24	27.41			
	SinoVac	No	441	446.13			
		Yes	27	21.86			
Number of doses	One	No	5126	5103.91	1	4.37	0.036
		Yes	229	250.08			
	Two	No	4466	4488.08			
		Yes	242	219.91			

χ^2^, chi-square; DF, degree of freedom; *p*, *p*-value (significant at ≤0.01).

**Table 6:** Accuracy and Cohen’s κ values for predicting the frequency and severity of post-vaccination side effects, based on 15 predisposing factors, using 11 ML tools.

Post-Vaccination Side Effects	ML Tools										
	XGBoost	RF	MLP	PNN	LibSVM (nu)	LibSVM (c)	AdaBoost	GB	KNN	K*	LWL
Tiredness	68 (32) *	68 (33) *	66 (27) *	59 (0)	59 (17)	59 (16)	66 (24) *	69 (33) *	62 (17)	66 (29) *	63 (23) *
Injection site pain and swelling	66 (29) *	66 (31) *	65 (27) *	58 (0)	59 (15)	58 (15)	64 (25) *	67 (31) *	61 (17)	66 (28) *	61 (20) *
Sleepiness and laziness	61 (22) *	63 (23) *	61 (21) *	54 (0)	56 (11)	56 (11)	62 (22) *	63 (25) *	59 (16)	62 (23) *	58 (17)
Headache	63 (25) *	64 (25) *	62 (22) *	55 (0)	55 (10)	56 (11)	61 (20)	64 (26) *	59 (16)	63 (24) *	59 (18)
Myalgia	65 (25) *	65 (23) *	64 (23) *	59 (0)	57 (11)	57 (11)	63 (20) *	66 (27) *	61 (16)	64 (23) *	62 (14)
Fever	67 (30) *	68 (28) *	66 (26) *	61 (0)	59 (14)	58 (14)	67 (26) *	69 (31) *	62 (18)	67 (28) *	65 (21)
Joint pain	66 (24) *	67 (21) *	66 (23) *	62 (0)	58 (11)	58 (12)	65 (18)	67 (26) *	63 (13)	65 (19)	64 (15)
Dizziness	71 (16)	72 (10)	72 (13)	72 (0)	62 (10)	62 (10)	72 (7)	72 (15)	70 (12)	71 (16)	72 (0)
Chills	78 (11)	79 (3)	79 (2)	79 (0)	67 (8)	66 (7)	79 (0)	79 (6)	77 (9)	78 (9)	79 (0)
Anxiety, depression and sleep disorders	72 (16)	73 (10)	73 (8)	73 (0)	61 (7)	61 (7)	73 (6)	73 (14)	71 (15)	72 (14)	73 (0)
Cold, numbness and tingling in limbs	73 (22) *	74 (16)	73 (17)	72 (0)	62 (10)	63 (13)	73 (9)	74 (22) *	70 (13)	72 (18)	72 (0)
Sweating for no reason	79 (7)	81 (2)	80 (2)	81 (0)	70 (7)	66 (5)	81 (0)	80 (4)	79 (8)	80 (7)	81 (0)
Sore or dry throat	81 (6)	82 (2)	82 (4)	82 (0)	70 (6)	70 (6)	82 (0)	82 (3)	80 (6)	81 (5)	82 (0)
Nausea	82 (8)	84 (2)	83 (1)	84 (0)	73 (11)	74 (8)	84 (0)	83 (4)	80 (8)	83 (7)	84 (0)
Irregular heartbeats	84 (8)	85 (2)	84 (6)	85 (0)	75 (9)	76 (10)	85 (0)	85 (5)	84 (8)	84 (9)	85 (0)
Abdominal pain	85 (6)	86 (1)	86 (1)	86 (0)	77 (9)	77 (8)	86 (0)	85 (1)	84 (9)	85 (7)	86 (0)
Clogged nose	86 (4)	87 (0)	86 (1)	87 (0)	77 (4)	78 (5)	87 (0)	87 (1)	86 (8)	86 (4)	87 (0)
Haziness or lack-of-clarity in eyesight	86 (7)	87 (2)	87 (0)	87 (0)	56 (11)	78 (8)	87 (0)	87 (0)	85 (8)	87 (7)	87 (0)
Dyspnea	87 (4)	88 (0)	88 (2)	88 (0)	81 (9)	81 (7)	88 (0)	88 (2)	87 (7)	88 (4)	88 (0)
Chest pain	88 (6)	88 (2)	88 (5)	88 (0)	80 (7)	81 (8)	88 (0)	88 (3)	87 (6)	88 (5)	88 (0)
Diarrhea	88 (2)	89 (1)	89 (2)	89 (0)	82 (6)	81 (5)	89 (0)	89 (1)	87 (5)	88 (5)	89 (0)
Runny nose	89 (4)	89 (1)	89 (2)	89 (0)	83 (6)	83 (6)	89 (0)	89 (0)	89 (5)	89 (5)	89 (0)
Cough	91 (7)	91 (1)	91 (2)	91 (0)	87 (8)	87 (8)	91 (0)	91 (3)	91 (6)	91 (7)	91 (0)
Abnormal blood pressure	91 (7)	92 (0)	91 (2)	92 (0)	88 (8)	88 (8)	92 (0)	92 (3)	91 (4)	91 (5)	92 (0)
Itchy skin, or irritation and allergic reactions	92 (4)	92 (1)	92 (1)	92 (0)	89 (8)	89 (8)	92 (0)	92 (3)	91 (6)	92 (5)	92 (0)
Swollen ankles and feet	95 (7)	95 (1)	95 (3)	95 (0)	93 (8)	93 (12)	95 (0)	95 (4)	95 (6)	95 (7)	95 (0)
Bruises on the body	95 (4)	92 (2)	95 (2)	95 (0)	93 (8)	93 (7)	95 (0)	95 (4)	95 (9)	95 (5)	95 (0)
Vomiting	96 (1)	96 (0)	96 (0)	96 (0)	95 (6)	94 (5)	96 (0)	96 (1)	96 (4)	96 (5)	96 (0)
Bleeding gums	98 (0)	99 (0)	98 (2)	99 (0)	98 (5)	98 (4)	99 (0)	98 (0)	98 (7)	98 (4)	99 (0)
Nosebleed	98 (4)	99 (0)	98 (2)	99 (0)	98 (4)	98 (6)	99 (0)	99 (1)	99 (5)	99 (5)	99 (0)
Severity of post-vaccination side effects	45 (17)	46 (17)	44 (13)	41 (0)	38 (11)	38 (11)	41 (5)	45 (17)	42 (12)	43 (15)	41 (6)

* The best-predicted side effects; Cohen’s κ > 20. XGBoost, eXtreme gradient boosting; RF, random forest; MLP, multilayer perceptron; PNN, probabilistic neural network (PNN); LibSVM, library for support vector machines (LibSVM), LibSVM (C) ranges from 0 to infinity; LibSVM (nu) ranges between 0 and 1; AdaBoost, adaptive boosting; GB, gradient boosting; KNN, K-nearest neighbor; K*, K-star; LWL, locally weighted learning.

**Table 7:** Feature importance values resulted from gradient boosting.

Predisposing Factors	Post-Vaccination Side Effects						
	Tiredness	Fever	Headache	Injection Site Pain and Swelling	Myalgia	Numbness and Tingling in Limbs	Sleepiness and Laziness
Gender	1.86	0.82	1.56	1.71	1.05	0.71	1.81
Age	1.25	1.53	1.28	1.43	1.55	1.08	1.83
Education level	0.63	0.71	0.58	1.05	0.75	0.91	0.64
Being a healthcare worker	0.23	0.18	0.11	0.32	0.08	0.14	0.18
Country	2.33	2.18	2.10	2.18	2.24	2.12	2.03
Suffering from chronic diseases	1.45	1.69	1.23	1.47	1.67	1.69	1.69
Being smoker	0.11	0.19	0.16	0.24	0.04	0.31	0.09
Suffering from food and/or drug allergies	0.17	0.51	0.11	0.43	0.40	0.20	0.46
Experiencing COVID-19 infection before receiving any vaccine dose	0.52	0.057	0.73	0.32	0.26	0.15	0.75
Experiencing COVID-19 vaccine hesitancy and related fears before vaccination	1.22	0.91	1.03	0.92	0.89	1.08	1.2
Type of COVID-19 vaccine	2.03	2.58	2.34	2.48	2.15	2.44	1.65
Interval between receiving a COVID-19 vaccine and participating in this study	0.60	0.66	0.59	0.27	0.54	0.56	0.57
Number of doses	0.77	1.13	0.83	0.28	0.80	0.34	0.24
Experiencing COVID-19 vaccine breakthrough infection	0.16	0.44	0.48	0.45	0.25	0.78	0.17
Time of breakthrough infection	0.90	1.01	1.48	0.90	1.16	1.67	0.64

Global feature importance was calculated using gradient boost (AutoML), which is used as standard pre-processing for training and optimizing ML tool. Surrogate RF was used to inspect global feature importance for the classification of each of the top ranked predicted symptoms in the previous table.

**Table 8:** Use of GLM/SRF to determine feature importance for different components of the critical features determined in Table S3.

Predisposing Factors		Post-Vaccination Side Effects											
		Tiredness		Fever		Headache		Injection Site Pain and Swelling		Myalgia		Sleepiness and Laziness	
		GLM	RF	GLM	RF	GLM	RF	GLM	RF	GLM	RF	GLM	RF
Number of doses	Two	9.93	0.6	-	-	7.1	0.93	-	-	3.72	0.91	-	-
Gender	Female	40.69	1.97	-	-	-	-	34.92	1.16	2.82	0.73	-	-
Experiencing COVID-19 vaccine hesitancy and related fears before vaccination	Yes	28.05	1.19	-	-	4.84	1.05	18.65	0.76	3.08	0.94	0.69	1.59
Type of COVID-19 vaccine	AstraZeneca	34.72	1.57	23.76	1.51	19.45	1.71	19.67	1.53	4.58	1.7	0.18	0.59
	Pfizer-BioNTech	−14.82	0.47	−0.17	1.15	4.3	0.74	0.1	0.81	−1.31	0.53	0.19	0.85
	Sinopharm	−22.12	0.38	−0.1	0.87	−6.71	1.4	−54.27	1.86	−10.67	1.32	−0.4	1.55
	Moderna	28.78	0.87	23.7	1.23	11.09	0.78	19.85	1.00	2.34	0.89	0.46	1.06
	Sputnik V	−3.82	0.17	−0.1	0.52	−6.46	1.05	−17.1	0.49	0.06	0.26	−0.35	1.16
	SinoVac	−28.84	0.62	−0.1	0.35	−6.16	0.91	−54.07	1.48	−8.76	0.74	−0.37	0.87
Age (years)	<20	-	-	-	-	-	-	-	-	−3.91	0.64	-	-
	20–39	-	-	-	-	-	-	-	-	−1.09	0.92	-	-
	40–59	-	-	-	-	-	-	-	-	−4.55	0.44	-	-
	>60	-	-	-	-	-	-	-	-	−11.29	0.92	-	-

GB is used as AutoML. GLM, generalized linear models; RF, random forest.

**Table 9:** List of studies on the side effects following COVID-19 vaccination in Arab countries.

Country	Population	Sample Size	Vaccines (%)	Reference
Iraq	General population	1012	AstraZeneca (60.1) Pfizer-BioNTech (29.2) Sinopharm (10.7)	[77]
Jordan	General population	2213	Sinopharm (38.2) AstraZeneca (31) Pfizer-BioNTech (27.3) Sputnik V (2.9) Moderna, Coaxin, and Johnson & Johnson (0.6)	[13]
Jordan	General population	1086	Sinopharm (26.4)	[78]
Jordan	General population	1004	Sinopharm (51.1) Pfizer-BioNTech (48.9)	[79]
Jordan	Healthcare workers	409	AstraZeneca (43.8) Pfizer-BioNTech (34.5) Sinopharm (21.8)	[80]
Kuwait	People with epilepsy	82	Pfizer-BioNTech (62) AstraZeneca (38)	[81]
Oman	General population	753	AstraZeneca (78) Pfizer-BioNTech (22)	[82]
Saudi Arabia	General population	18,543	AstraZeneca (97.8) Pfizer-BioNTech (2.3)	[83]
Saudi Arabia	General population	4170	Pfizer-BioNTech (61) AstraZeneca (39)	[84]
Saudi Arabia	General population	1592	AstraZeneca	[85]
Saudi Arabia	General population	515	AstraZeneca (75) Pfizer-BioNTech (25)	[86]
Saudi Arabia	General population	455	Pfizer-BioNTech	[87]
Saudi Arabia	General population	330	AstraZeneca (50.6) Pfizer-BioNTech (49.4)	[88]
UAE	General population	1080	Sinopharm	[12]

UAE, United Arab Emirates.

**Table 10:** Characterization of participants who experienced thrombosis following COVID-19 vaccination (*n* = 22). All of them were admitted to hospitals and needed more than one week to recover.

Gender	Age Category (Year)	Country	Chronic Diseases	Smoking Status	Vaccine	Dose	Interval between Receiving a COVID-19 Vaccine and Thrombosis	Thrombocytopenia	Causes of Hospitalization/Type of Thrombosis (If Known)
Female	20–39	Egypt	Arthritis	No	AstraZeneca	1	12–24 h	Yes	Cerebral venous thrombosis
Female	40–59	Egypt	Autoimmune diseases	No	AstraZeneca	1	Up to 4 h	Yes	Chest pain and dyspnea
Female	40–59	Egypt	Diabetes	No	AstraZeneca	1	More than 24 h	No	Chest pain and dyspnea
Male	20–39	Saudi Arabia	Obesity	No	AstraZeneca	1	More than 24 h	No	Chest pain and dyspnea
Female	40–59	Algeria	Thyroid disorders	No	AstraZeneca	1	Up to 4 h	Yes	-
Female	40–59	Algeria	-	No	AstraZeneca	1	More than 24 h	No	Numbness and tingling in the limbs, palpitation and hypertension
Female	20–39	Jordan	-	No	AstraZeneca	1	5–12 h	No	Chest pain
Male	More than 60	Egypt	Obesity and hypertension	No	AstraZeneca	1	More than 24 h	No	-
Male	20–39	Saudi Arabia	-	No	AstraZeneca	2	5–12 h	Yes	Cerebral venous thrombosis
Female	20–39	Algeria	Hypertension	No	AstraZeneca	2	More than 24 h	Yes	Chest pain and hypoxemia
Female	40–59	Egypt	Autoimmune diseases and hypertension	No	AstraZeneca	2	Up to 4 h	Yes	Chest pain and dyspnea
Male	20–39	Jordan	-	Yes	AstraZeneca	2	More than 24 h	Yes	Fever
Female	20–39	Jordan	Respiratory diseases	No	AstraZeneca	2	5–12 h	No	Chest pain, headache, blurry vision and dyspnea
Female	40–59	Jordan	Obesity, diabetes, cardiovascular diseases, thyroid disorders	No	Pfizer-BioNTech	1	Up to 4 h	No	Deep vein thrombosis in the leg, dyspnea, tachycardia and vomiting
Female	40–59	Tunisia	Arthritis	No	Pfizer-BioNTech	1	12–24 h	No	Numbness in the left side of the body and hypertension
Male	40–59	Jordan	Diabetes, hypertension and obesity	Yes	Pfizer-BioNTech	1	More than 24 h	No	Pulmonary embolism and unconsciousness
Female	More than 60	Saudi Arabia	Hypertension	No	Pfizer-BioNTech	1	More than 24 h	No	Cerebral venous thrombosis
Male	20–39	Iraq	-	Yes	Pfizer-BioNTech	1	12–24 h	Yes	-
Male	20–39	Jordan	-	No	Pfizer-BioNTech	2	More than 24 h	No	Supraventricular tachycardia and elevated cardiac enzymes
Male	20–39	Iraq	Obesity	Yes	Pfizer-BioNTech	2	More than 24 h	No	-
Male	More than 60	Jordan	Diabetes	Yes	Pfizer-BioNTech	2	5–12 h	Yes	Dizziness
Female	More than 60	Tunisia	Hypertension, obesity, diabetes, cardiovascular diseases, thyroid disorders and arthritis	Yes	Johnson & Johnson	1	Up to 4 h	Yes	Chest pain and dyspnea

## Data Availability

All data generated are contained in the present manuscript.

## Supplementary Materials

The following supporting information can be downloaded at: https://www.mdpi.com/article/10.3390/vaccines10030366/s1, Survey of Side Effects and Perceptions Following COVID-19 Vaccination in the Arab World; Table S1: The observed frequencies that used in performing the χ^2^ test; Table S2: Correlation between categories of predisposing factors; Table S3: The accuracy and Cohen’s values for different top predicted symptoms using backward elimination method from the least to the highest global feature importance to determine the steepest decline in prediction (GB was used as predicting ML tool).
