# Supplementary material for: Reported Adverse Effects and Attitudes among Arab Populations Following COVID-19 Vaccination: A Large-Scale Multinational Study Implementing Machine Learning Tools in Predicting Post-Vaccination Adverse Effects Based on Predisposing Factors
Source: Vaccines (Basel). 2022 Feb 26;10(3):366. doi: 10.3390/vaccines10030366 (PMC8955470; doi:10.3390/vaccines10030366)
Supplement: Supplementary file 1 [file vaccines-10-00366-s001.zip › Supplementary Tables.pdf]

Supplementary Materials

Table S1. The observed frequencies that used in performing the χ2 test.

Predisposing factors	Post-vaccination side effects																	
	Tiredness	Anxiety, depression and sleep disorders	Fever	Headache	Haziness or lack-of-clarity in eyesight	Injection site pain and swelling	Joints pain	Swollen ankles and feet	Myalgia	Nausea	Abdominal pain	Diarrhea	Vomiting	Bruises on the body	Bleeding gums	Nosebleed	Chills	Itchy skin, or irritation and allergic reactions
**Gender**																		
Male	0.49	0.22	0.35	0.36	0.1	0.47	0.3	0.02	0.34	0.1	0.1	0.09	0.03	0.02	0.01	0.01	0.21	0.06
Female	0.67	0.31	0.43	0.52	0.15	0.67	0.43	0.07	0.47	0.22	0.18	0.13	0.05	0.06	0.02	0.01	0.33	0.1
**Age** (years)																		
< 20	0.46	0.19	0.25	0.37	0.08	0.5	0.22	0.01	0.29	0.13	0.13	0.07	0.03	0.02	0.01	0.02	0.15	0.05
20 - 39	0.64	0.3	0.43	0.48	0.14	0.63	0.4	0.04	0.45	0.19	0.17	0.13	0.05	0.05	0.02	0.01	0.3	0.08
40 - 59	0.55	0.25	0.38	0.43	0.13	0.53	0.38	0.06	0.39	0.14	0.11	0.1	0.03	0.04	0.02	0.01	0.27	0.08
> 60	0.38	0.15	0.29	0.28	0.08	0.39	0.24	0.05	0.26	0.09	0.06	0.07	0.02	0.03	0.01	0.02	0.17	0.06
**Education level**																		
High school or less	0.52	0.25	0.36	0.41	0.13	0.5	0.35	0.06	0.35	0.15	0.14	0.11	0.04	0.04	0.02	0.02	0.23	0.08
Undergraduate	0.61	0.29	0.41	0.47	0.13	0.61	0.39	0.04	0.44	0.18	0.15	0.12	0.04	0.05	0.01	0.01	0.29	0.08
Postgraduate	0.56	0.24	0.37	0.41	0.11	0.55	0.35	0.04	0.38	0.14	0.13	0.1	0.03	0.04	0.02	0.01	0.27	0.08
**Being healthcare worker**																		
Yes	0.6	0.26	0.42	0.45	0.11	0.6	0.36	0.03	0.42	0.15	0.12	0.09	0.04	0.04	0.01	0.02	0.28	0.07
No	0.58	0.28	0.38	0.45	0.14	0.57	0.38	0.05	0.41	0.17	0.15	0.12	0.04	0.05	0.02	0.01	0.27	0.09
**Country**																		
Jordan	0.67	0.35	0.4	0.51	0.19	0.62	0.46	0.09	0.48	0.21	0.19	0.16	0.04	0.08	0.03	0.03	0.37	0.11

Lebanon	0.52	0.18	0.25	0.38	0.08	0.54	0.26	0.02	0.33	0.13	0.12	0.09	0.03	0.02	0.01	0.01	0.17	0.04
Saudi Arabia	0.59	0.23	0.4	0.43	0.11	0.63	0.35	0.04	0.42	0.13	0.09	0.07	0.03	0.04	0.02	0.01	0.23	0.07
Iraq	0.67	0.34	0.59	0.55	0.15	0.72	0.5	0.03	0.51	0.2	0.16	0.11	0.04	0.05	0.02	0.01	0.31	0.09
Egypt	0.76	0.41	0.55	0.58	0.23	0.67	0.48	0.07	0.56	0.22	0.22	0.17	0.07	0.08	0.02	0.01	0.4	0.12
Palestine	0.67	0.32	0.48	0.53	0.12	0.63	0.46	0.05	0.47	0.19	0.15	0.13	0.06	0.04	0.02	0.02	0.42	0.06
Tunisia	0.5	0.26	0.36	0.39	0.09	0.54	0.36	0.05	0.29	0.12	0.12	0.07	0.04	0.02	0.02	0.01	0.26	0.06
Qatar	0.69	0.24	0.51	0.52	0.08	0.67	0.46	0.02	0.47	0.19	0.16	0.1	0.04	0.03	0.01	0	0.41	0.1
Kuwait	0.74	0.32	0.49	0.54	0.17	0.69	0.49	0.05	0.52	0.19	0.2	0.16	0.03	0.07	0.01	0.01	0.39	0.1
Algeria	0.42	0.23	0.36	0.35	0.12	0.37	0.3	0.04	0.29	0.14	0.15	0.14	0.04	0.02	0.01	0.01	0.2	0.08
Libya	0.47	0.25	0.36	0.37	0.09	0.43	0.34	0.04	0.31	0.14	0.12	0.11	0.03	0.04	0	0	0.22	0.06
Syria	0.33	0.17	0.3	0.23	0.07	0.28	0.19	0.03	0.21	0.11	0.08	0.06	0.01	0.03	0.02	0.04	0.19	0.09
Morocco	0.49	0.25	0.35	0.35	0.13	0.41	0.33	0.02	0.35	0.19	0.15	0.13	0.06	0.04	0.02	0.02	0.28	0.06
Bahrain	0.38	0.17	0.22	0.3	0.06	0.4	0.25	0.03	0.28	0.13	0.06	0.03	0.04	0.04	0.01	0	0.09	0.07
UAE	0.46	0.16	0.21	0.36	0.08	0.45	0.29	0.06	0.3	0.13	0.11	0.1	0.01	0.04	0.02	0.01	0.24	0.07
Oman	0.57	0.18	0.29	0.5	0.11	0.55	0.3	0.04	0.42	0.12	0.04	0.05	0.03	0.04	0.03	0.01	0.21	0.04
Sudan	0.6	0.27	0.41	0.4	0.13	0.7	0.37	0.02	0.37	0.13	0.14	0.1	0.02	0.03	0	0.02	0.17	0.05
Yemen	0.56	0.12	0.38	0.42	0.1	0.54	0.3	0.02	0.4	0.14	0.14	0.08	0.02	0.02	0.02	0.04	0.32	0.08
Mauritania	0.55	0.18	0.36	0.36	0	0.36	0.36	0	0.36	0.18	0.18	0	0.09	0.09	0	0	0.36	0
**Type of COVID-19 vaccine**																		
AstraZeneca	0.73	0.36	0.62	0.58	0.17	0.67	0.52	0.06	0.55	0.21	0.17	0.14	0.05	0.06	0.02	0.02	0.43	0.1
Pfizer-BioNTech	0.58	0.25	0.35	0.44	0.11	0.63	0.35	0.04	0.4	0.15	0.13	0.1	0.03	0.04	0.01	0.01	0.24	0.07
Sinopharm	0.61	0.3	0.42	0.46	0.15	0.59	0.42	0.06	0.44	0.2	0.17	0.12	0.05	0.06	0.01	0.02	0.32	0.1
Johnson & Johnson	0.59	0.24	0.31	0.47	0.17	0.53	0.33	0.05	0.29	0.14	0.12	0.07	0.02	0.03	0.02	0.03	0.22	0.05
Moderna	0.54	0.24	0.36	0.4	0.14	0.5	0.38	0.04	0.36	0.17	0.11	0.06	0.06	0.06	0.02	0.01	0.29	0.07
Sputnik V	0.61	0.3	0.42	0.46	0.14	0.58	0.4	0.06	0.42	0.17	0.15	0.13	0.05	0.05	0.02	0.02	0.29	0.1
SinoVac	0.59	0.27	0.42	0.48	0.13	0.56	0.36	0.04	0.39	0.17	0.15	0.12	0.04	0.05	0.02	0.01	0.25	0.08
**Number of doses**																		
One	0.57	0.28	0.36	0.42	0.13	0.57	0.35	0.05	0.39	0.17	0.15	0.12	0.04	0.05	0.01	0.01	0.24	0.08

Two	0.61	0.26	0.43	0.48	0.12	0.6	0.41	0.04	0.44	0.16	0.14	0.11	0.04	0.05	0.02	0.02	0.32	0.08
**Suffering from chronic diseases**																		
No	0.82	0.37	0.54	0.62	0.16	0.82	0.5	0.05	0.56	0.23	0.19	0.14	0.05	0.06	0.02	0.02	0.37	0.1
Diabetes	0.21	0.09	0.15	0.16	0.04	0.21	0.14	0.01	0.15	0.07	0.05	0.03	0.02	0.01	0.01	0	0.12	0.02
Hypertension	0.28	0.12	0.19	0.2	0.07	0.27	0.16	0.03	0.18	0.07	0.07	0.05	0.02	0.02	0.01	0	0.13	0.03
Cardiovascular	0.25	0.13	0.16	0.2	0.08	0.26	0.16	0.03	0.19	0.09	0.08	0.05	0.02	0.02	0	0	0.13	0.03
Respiratory disease	0.37	0.16	0.25	0.28	0.09	0.34	0.26	0.03	0.25	0.11	0.1	0.09	0.03	0.03	0	0.01	0.17	0.06
Obesity	0.36	0.15	0.24	0.25	0.08	0.33	0.21	0.03	0.24	0.09	0.09	0.06	0.03	0.03	0.01	0.01	0.18	0.03
Arthritis	0.27	0.13	0.2	0.22	0.06	0.24	0.17	0.02	0.17	0.08	0.04	0.03	0.01	0.02	0	0.01	0.12	0.04
Autoimmune diseases	0.38	0.19	0.22	0.27	0.08	0.34	0.22	0.04	0.22	0.1	0.07	0.06	0.01	0.03	0.01	0	0.16	0.03
Thyroid dysfunctions	0.36	0.13	0.25	0.24	0.08	0.35	0.22	0.03	0.25	0.1	0.09	0.07	0.02	0.02	0.01	0.01	0.19	0.04
Cancers	0.37	0.2	0.14	0.24	0.08	0.39	0.2	0.04	0.22	0.08	0.06	0.04	0.04	0.02	0.02	0.02	0.14	0.04
More than one disease	0.6	0.25	0.38	0.44	0.13	0.57	0.4	0.04	0.43	0.17	0.15	0.12	0.04	0.04	0.01	0.02	0.27	0.08
**Being smoker**																		
Yes	0.57	0.28	0.38	0.43	0.14	0.56	0.38	0.04	0.42	0.15	0.14	0.11	0.04	0.05	0.02	0.02	0.28	0.08
No	0.6	0.27	0.4	0.45	0.12	0.59	0.37	0.05	0.41	0.17	0.14	0.11	0.04	0.05	0.01	0.01	0.28	0.08
**Suffering from food and drug allergies**																		
Yes	0.63	0.31	0.44	0.52	0.16	0.64	0.43	0.07	0.46	0.22	0.18	0.13	0.06	0.07	0.03	0.02	0.32	0.14
No	0.58	0.27	0.39	0.44	0.12	0.57	0.37	0.04	0.41	0.16	0.14	0.11	0.04	0.04	0.01	0.01	0.27	0.07
**Experiencing COVID-19 vaccine hesitancy and related fears before vaccination**																		
Yes	0.66	0.35	0.44	0.51	0.17	0.64	0.44	0.07	0.47	0.21	0.18	0.14	0.05	0.06	0.02	0.02	0.33	0.1
No	0.52	0.19	0.35	0.38	0.08	0.52	0.31	0.02	0.35	0.12	0.1	0.08	0.03	0.03	0.01	0.01	0.22	0.05
**Experiencing COVID-19 infection before receiving any vaccine dose**																		
Yes	0.65	0.27	0.39	0.44	0.14	0.58	0.38	0.05	0.42	0.17	0.14	0.11	0.04	0.05	0.02	0.02	0.27	0.08
No	0.57	0.27	0.39	0.45	0.12	0.58	0.37	0.05	0.41	0.16	0.14	0.11	0.04	0.04	0.01	0.01	0.28	0.08

Predisposing factors	Post-vaccination side effects												Severity of post-vaccination side effects			
	Sweating for no reason	Cold, numbness and tingling in limbs	Dizziness	Clogged nose	Runny nose	Dyspnea	Chest pain	Sleepiness and laziness	Irregular heartbeats	Abnormal blood pressure	Sore or dry throat	Cough	None	Mild	Moderate	Severe
**Gender**																
Male	0.16	0.16	0.2	0.11	0.08	0.09	0.1	0.37	0.11	0.06	0.13	0.07	0.36	0.38	0.18	0.07
Female	0.21	0.24	0.35	0.15	0.12	0.14	0.13	0.53	0.19	0.1	0.21	0.1	0.13	0.43	0.26	0.11
**Age**																
< 20	0.14	0.14	0.23	0.1	0.08	0.1	0.07	0.36	0.09	0.04	0.12	0.05	0.42	0.42	0.13	0.03
20 - 39	0.2	0.23	0.32	0.15	0.12	0.13	0.13	0.51	0.17	0.09	0.2	0.1	0.24	0.41	0.24	0.11
40 - 59	0.19	0.19	0.25	0.12	0.09	0.1	0.1	0.42	0.14	0.08	0.16	0.07	0.29	0.4	0.22	0.09
> 60	0.13	0.12	0.17	0.08	0.09	0.06	0.05	0.28	0.08	0.07	0.13	0.08	0.4	0.41	0.14	0.06
**Education level**																
High school or less	0.2	0.2	0.24	0.13	0.11	0.12	0.12	0.41	0.14	0.09	0.18	0.1	0.33	0.39	0.2	0.08
Undergraduate	0.2	0.22	0.3	0.14	0.11	0.13	0.12	0.48	0.16	0.08	0.19	0.09	0.26	0.41	0.23	0.1
Postgraduate	0.17	0.18	0.26	0.11	0.09	0.1	0.1	0.42	0.13	0.07	0.15	0.07	0.29	0.41	0.21	0.09
**Being healthcare worker**																
Yes	0.17	0.19	0.26	0.13	0.1	0.1	0.1	0.44	0.14	0.07	0.15	0.08	0.27	0.41	0.22	0.09
No	0.2	0.21	0.29	0.13	0.11	0.12	0.12	0.47	0.16	0.09	0.19	0.09	0.28	0.41	0.22	0.09
**Country**																
Jordan	0.23	0.26	0.35	0.18	0.13	0.18	0.18	0.54	0.2	0.12	0.26	0.11	0.24	0.4	0.24	0.11

Lebanon	0.13	0.14	0.22	0.1	0.07	0.08	0.06	0.41	0.08	0.04	0.12	0.06	0.36	0.45	0.15	0.04
Saudi Arabia	0.15	0.18	0.22	0.09	0.09	0.09	0.08	0.42	0.12	0.06	0.13	0.05	0.24	0.43	0.24	0.09
Iraq	0.24	0.27	0.39	0.17	0.16	0.14	0.16	0.56	0.21	0.13	0.22	0.12	0.15	0.43	0.28	0.13
Egypt	0.24	0.28	0.45	0.21	0.15	0.19	0.18	0.6	0.29	0.16	0.26	0.11	0.15	0.35	0.32	0.19
Palestine	0.25	0.24	0.3	0.14	0.1	0.13	0.13	0.48	0.17	0.09	0.2	0.12	0.24	0.38	0.27	0.11
Tunisia	0.25	0.18	0.23	0.12	0.1	0.05	0.09	0.39	0.13	0.05	0.18	0.09	0.29	0.43	0.22	0.06
Qatar	0.22	0.24	0.29	0.09	0.08	0.1	0.08	0.49	0.11	0.05	0.14	0.05	0.19	0.4	0.29	0.13
Kuwait	0.23	0.33	0.38	0.14	0.12	0.16	0.17	0.6	0.15	0.1	0.2	0.13	0.16	0.41	0.13	0
Algeria	0.22	0.19	0.23	0.14	0.09	0.11	0.12	0.35	0.16	0.07	0.18	0.08	0.4	0.36	0.18	0.07
Libya	0.17	0.18	0.23	0.11	0.08	0.08	0.07	0.34	0.11	0.05	0.18	0.06	0.4	0.35	0.18	0.07
Syria	0.17	0.13	0.16	0.11	0.11	0.09	0.07	0.25	0.11	0.06	0.1	0.09	0.6	0.21	0.13	0.06
Morocco	0.2	0.2	0.3	0.14	0.08	0.14	0.14	0.45	0.19	0.1	0.17	0.12	0.33	0.36	0.24	0.07
Bahrain	0.1	0.12	0.17	0.06	0.07	0.07	0.06	0.35	0.04	0.06	0.08	0.04	0.42	0.37	0.15	0.06
UAE	0.1	0.17	0.18	0.07	0.06	0.05	0.12	0.33	0.08	0.05	0.13	0.06	0.38	0.43	0.15	0.04
Oman	0.11	0.18	0.24	0.09	0.07	0.07	0.07	0.39	0.07	0.01	0.17	0.01	0.17	0.59	0.16	0.08
Sudan	0.08	0.14	0.22	0.11	0.11	0.13	0.1	0.46	0.13	0.02	0.08	0.06	0.22	0.51	0.19	0.08
Yemen	0.16	0.3	0.28	0.12	0.14	0.08	0.12	0.44	0.06	0.06	0.14	0.12	0.28	0.48	0.24	0
Mauritania	0.18	0.09	0.18	0.18	0	0.09	0.09	0.36	0.09	0.09	0.09	0.09	0.27	0.55	0.09	0.09
**Type of COVID-19 vaccine**																
AstraZeneca	0.26	0.3	0.37	0.16	0.12	0.15	0.16	0.54	0.21	0.12	0.22	0.11	0.16	0.31	0.33	0.2
Pfizer-BioNTech	0.17	0.18	0.26	0.12	0.1	0.11	0.1	0.45	0.13	0.07	0.16	0.08	0.27	0.45	0.21	0.07
Sinopharm	0.23	0.24	0.32	0.15	0.11	0.14	0.15	0.5	0.18	0.1	0.21	0.11	0.39	0.43	0.15	0.03
Johnson & Johnson	0.26	0.19	0.24	0.12	0.16	0.09	0.12	0.41	0.16	0.05	0.1	0.07	0.05	0.24	0.45	0.26
Moderna	0.17	0.19	0.24	0.17	0.12	0.12	0.14	0.41	0.16	0.05	0.17	0.08	0.16	0.32	0.3	0.22
Sputnik V	0.21	0.21	0.31	0.14	0.11	0.13	0.11	0.47	0.18	0.1	0.19	0.11	0.35	0.37	0.22	0.06
SinoVac	0.21	0.21	0.29	0.15	0.11	0.13	0.13	0.47	0.15	0.09	0.2	0.09	0.46	0.35	0.13	0.06
**Number of doses**																
One	0.19	0.21	0.29	0.13	0.11	0.12	0.12	0.45	0.15	0.08	0.19	0.09	0.29	0.41	0.2	0.09

Two	0.19	0.21	0.28	0.13	0.11	0.11	0.11	0.47	0.15	0.08	0.17	0.08	0.25	0.4	0.25	0.1
**Suffering from chronic diseases**																
No	0.25	0.28	0.39	0.17	0.13	0.16	0.15	0.64	0.19	0.1	0.23	0.11	0.4	0.57	0.31	0.12
Diabetes	0.08	0.07	0.11	0.04	0.04	0.04	0.05	0.19	0.06	0.02	0.07	0.03	0.13	0.15	0.06	0.03
Hypertension	0.09	0.09	0.14	0.06	0.04	0.05	0.05	0.22	0.07	0.03	0.08	0.04	0.11	0.18	0.11	0.05
Cardiovascular	0.09	0.1	0.14	0.03	0.04	0.06	0.05	0.19	0.1	0.05	0.07	0.03	0.12	0.15	0.08	0.04
Respiratory diseases	0.11	0.12	0.18	0.09	0.07	0.07	0.07	0.29	0.09	0.05	0.12	0.06	0.11	0.25	0.2	0.05
Obesity	0.12	0.11	0.17	0.08	0.06	0.06	0.06	0.28	0.06	0.04	0.11	0.05	0.12	0.24	0.13	0.06
Arthritis	0.1	0.09	0.12	0.05	0.03	0.04	0.05	0.19	0.07	0.06	0.08	0.04	0.11	0.19	0.1	0.06
Autoimmunity	0.09	0.12	0.17	0.07	0.07	0.08	0.07	0.31	0.12	0.04	0.11	0.05	0.17	0.24	0.13	0.1
Thyroid dysfunctions	0.12	0.11	0.17	0.07	0.08	0.07	0.08	0.3	0.1	0.05	0.13	0.05	0.11	0.25	0.16	0.06
Cancers	0.1	0.14	0.14	0.06	0.08	0.04	0.1	0.27	0.1	0.08	0.1	0.06	0.08	0.29	0.16	0.06
More than one disease	0.2	0.21	0.29	0.13	0.11	0.1	0.11	0.46	0.15	0.09	0.18	0.09	0.26	0.39	0.24	0.11
**Being smoker**																
Yes	0.22	0.21	0.27	0.14	0.11	0.12	0.12	0.47	0.15	0.08	0.18	0.08	0.3	0.39	0.22	0.09
No	0.18	0.2	0.29	0.13	0.11	0.12	0.11	0.46	0.15	0.08	0.18	0.09	0.27	0.41	0.23	0.09
**Suffering from food and drug allergies**																
Yes	0.24	0.26	0.33	0.17	0.14	0.16	0.16	0.51	0.21	0.11	0.24	0.12	0.23	0.4	0.24	0.13
No	0.18	0.2	0.28	0.13	0.1	0.11	0.11	0.45	0.14	0.08	0.17	0.08	0.28	0.41	0.22	0.09
**Experiencing COVID-19 vaccine hesitancy and related fears before vaccination**																
Yes	0.23	0.25	0.35	0.16	0.13	0.15	0.15	0.53	0.2	0.11	0.22	0.11	0.22	0.41	0.26	0.11
No	0.16	0.16	0.21	0.11	0.09	0.08	0.08	0.39	0.1	0.05	0.13	0.07	0.33	0.41	0.18	0.07
**Experiencing COVID-19 infection before receiving any vaccine dose**																
Yes	0.19	0.21	0.28	0.13	0.11	0.13	0.13	0.46	0.16	0.09	0.18	0.09	0.23	0.42	0.25	0.1
No	0.19	0.21	0.28	0.13	0.11	0.11	0.11	0.46	0.15	0.08	0.18	0.08	0.29	0.4	0.21	0.09

Table S2. Correlation between categories of predisposing factors.

	Gender		Age (year)				Country															Vaccine hesitancy and related fears		Type of vaccine						Number of doses		Education level		
	Male	Female	< 20	20 – 39	40 – 59	> 60	Jordan	Egypt	Saudi Arabia	Iraq	Lebanon	Palestine	Morroco	Tunisia	Syria	Algeria	Qatar	Bahrain	Kuwait	UAE	Libya	No	Yes	AstraZeneca	Pfizer-BioNTech	Sinopharm	Moderna	Sputnik V	SinoVac	One	Two	Secondary school or less	Undergraduate	Postgraduate
**Gender**																																		
Male	1																																	
Female	-1	1																																
**Age (year)**																																		
< 20	-2	2	1																															
20 – 39	-5	5	-32	1																														
40 – 59	4	-4	-17	-77	1																													
> 60	5	-5	-6	-27	-15	1																												
**Country**																																		
Jordan	-13	13	-9	11	-4	-6	1																											
Egypt	-2	2	-7	1	4	-2	-13	1																										
Saudi Arabia	-2	2	-8	-5	7	6	-19	-12	1																									
Iraq	6	-6	-2	6	-3	-4	-14	-9	-14	1																								
Lebanon	-1	1	37	1	-18	-6	-22	-14	-21	-16	1																							
Palestine	-4	4		4	-4	-2	-11	-7	-1	-8	-12	1																						

Morroco	3	-3	-3	2	-1		-6	-4	-6	-5	-7	-3	1																					
Tunisia	-1	1	-5	-11	1	9	-9	-6	-8	-6	-1	-5	-3	1																				
Syria	11	-11	-4	-7	6	8	-8	-5	-8	-6	-9	-4	-3	-4	1																			
Algeria	5	-5	-5	-1	4	0	-9	-6	-9	-7	-1	-5	-3	-4	-4	1																		
Qatar	5	-5	-4	-2	4	-1	-7	-5	-7	-5	-8	-4	-2	-3	-3	-3	1																	
Bahrain	-2	2	-3	-2		8	-6	-4	-6	-4	-7	-3	-2	-3	-3	-3	-2	1																
Kuwait	3	-3	-4	-3	6	-2	-7	-4	-7	-5	-8	-4	-2	-3	-3	-3	-3	-2	1															
UAE	3	-3	-2	-2	4	-1	-5	-3	-5	-3	-5	-3	-1	-2	-2	-2	-2	-1	-2	1														
Libya	3	-3	-4	-8	7	7	-8	-5	-8	-6	-9	-4	-3	-4	-3	-4	-3	-2	-3	-2	1													
**Vaccine hesitancy and related fears**																																		
No	15	-15	9	-2	-4	3	-9	-9	-4	1	15	3	-3	1	2	-4	1	1	-1	1	1	1												
Yes	-15	15	-9	2	4	-3	9	9	4	-1	-15	-3	3	-1	-2	4	-1	-1	1	-1	-1	-1	1											
**Type of vaccine**																																		
AstraZeneca	9	-9	-1	-6	1	4	-1	3	7	14	-22	-8	2	-7	13	-2	-8	-6	11	-5	-2	-2	2	1										
Pfizer-BioNTech	-6	6	18	2	-12	-2	-5	-29	17	-5	4	5	-14	4	-19	-21	5	-8	-1	-4	-17	6	-6	-54	1									
Sinopharm	-7	7	-8	5	-1	-2	33	7	-17	1	-2	-6	2	-4	-6	-4	-6	19	-6	14	-6	-5	5	-21	-43	1								
Moderna	1	-1	-3	2	0	-1	-5	-2	-4	-4	-5	5	-2	4	-2	-2	46*	-1	-2	-1	-2	0	0	-6	-12	-4	1							
Sputnik V	4	-4	-5	-2	4	0	-1	-4	-11	-8	-4	14	-4	-2	23	5	-4	1	-4	-2	42*	1	-1	-13	-26	-1	-3	1						
SinoVac	3	-3	-4	-3	4	2	-9	3	-9	-7	-11	-4	0	4	6	57*	-4	0	-3	-2	8	-3	3	-11	-23	-9	-2	-5	1					
**Number of doses**																																		
One	-2	2	16	8	-13	-1	-8	6	4	-4	19	-13	-2	3	-11	6	-15	-12	-4	-6	14	-8	8	4	5	-14	-5	-1	5	1				
Two	2	-2	-16	-8	13	1	8	-6	-4	4	-19	13	2	-3	11	-6	15	12	4	6	-14	8	-8	-4	-5	14	5	1	-5	-1	1			
**Education level**																																		
Secondary school or less	2	-2	8	-15	6	11	-1	-6	3	1	-2	-4	-2	6	1	3	-3	2	2	2	1	-1	1	0	1	-1	-1	-3	3	5	-5	1		
Undergraduate	-7	7	4	15	-13	-11	8	3	0	8	4	8	-9	-17	-8	-18	3	-1	2	-2	1	0	0	0	7	0	2	-3	-13	5	-5	-46	1	

Postgraduate	6	-6	-1	-6	1	4	-8	0	-2	-1	-3	-6	12	15	8	18	-1	-1	-4	1	-2	0	0	0	-9	1	-2	5	12	-9	9	-21	-77	1

*Correlation between different categories from different factors with │R│ > 40 (indicated in yellow). Numbers written as n ×100. UAE, United Arab Emirates.

Table S3. The accuracy and Cohen's values for different top predicted symptoms using backward elimination method from the least to the highest global feature importance to determine the steepest decline in prediction (GB was used as predicting ML tool).

**Tiredness**	**FI**	**A**	**C**	**Fever**	**FI**	**A**	**C**
Being smoker	0.11	69	33	Experiencing vaccine breakthrough COVID-19 infection	0.057	69	31
Experiencing vaccine breakthrough COVID-19 infection	0.16	69	33	Being healthcare worker	0.18	69	32
Suffering from food and drug allergies	0.17	68	33	Being smoker	0.19	69	31
Being healthcare worker	0.23	69	34	Experiencing COVID-19 infection before receiving any vaccine dose	0.44	69	31
Experiencing COVID-19 infection before receiving any vaccine dose	0.52	68	33	Suffering from food and drug allergies	0.51	68	31
Interval between receiving a COVID-19 vaccine and participating in this study	0.6	68	32	Interval between receiving a COVID-19 vaccine and participating in this study	0.66	68	31
Education level	0.63	68	32	Education level	0.71	69	31
Number of doses **^	0.77	68	32	Gender	0.82	68	31
Time of breakthrough infection	0.9	66	29	Interval between receiving a COVID-19 vaccine and participating in this study **	0.91	68	30
Experiencing COVID-19 vaccine hesitancy and related fears before vaccination **^	1.22	67	29	Time of breakthrough infection	1.01	67	28
Age	1.25	66	27	Number of doses	1.13	67	27
Suffering from chronic diseases	1.45	66	27	Age	1.53	67	26
Gender **^	1.86	66	27	Suffering from chronic diseases	1.69	67	27
Type of COVID-19 vaccine **^	2.03	63	16	Country **^	2.18	68	27
Country **^	2.33	61	12	Type of COVID-19 vaccine **^	2.58	66	23

Critical features		68	32	Critical features		67	27
**Headache**	**FI**	**A**	**C**	**Injection site pain and swelling**	**FI**	**A**	**C**
Being healthcare worker	0.11	64	26	Being smoker	0.24	67	31
Suffering from food and drug allergies	0.11	64	26	Interval between receiving a COVID-19 vaccine and participating in this study	0.27	67	31
Being smoker	0.16	64	26	Number of doses	0.28	67	31
Experiencing vaccine breakthrough COVID-19 infection	0.48	64	27	Being healthcare worker	0.32	67	30
Education level	0.58	64	26	Experiencing COVID-19 infection before receiving any vaccine dose	0.32	67	30
Interval between receiving a COVID-19 vaccine and participating in this study	0.59	64	26	Suffering from food and drug allergies	0.43	67	29
Experiencing COVID-19 infection before receiving any vaccine dose	0.73	64	27	Experiencing vaccine breakthrough COVID-19 infection	0.45	66	29
Number of doses **^	0.83	64	27	Time of breakthrough infection	0.9	66	28
Experiencing COVID-19 vaccine hesitancy and related fears before vaccination **^	1.03	63	24	Experiencing COVID-19 vaccine hesitancy and related fears before vaccination **	0.92	67	30
Suffering from chronic diseases	1.23	63	22	Education level	1.05	66	28
Age	1.28	62	21	Age	1.43	66	28
Time of breakthrough infection	1.48	62	21	Suffering from chronic diseases	1.47	65	26
Gender	1.56	62	21	Gender**^	1.71	66	27
Country **^	2.1	61	20	Country**^	2.18	64	22
Type of COVID-19 vaccine **^	2.34	59	13	Type of COVID-19 vaccine **^	2.48	63	18
Critical features		62	23	Critical features		66	27
**Myalgia**	**FI**	**A**	**C**	**Numbness and tingling in limbs**	**FI**	**A**	**C**
Being smoker	0.04	66	27	Being healthcare worker	0.14	74	23
Being healthcare worker	0.08	66	27	Experiencing COVID-19 infection before receiving any vaccine dose	0.15	74	23

Experiencing vaccine breakthrough COVID-19 infection	0.25	65	26
Experiencing COVID-19 infection before receiving any vaccine dose	0.26	65	26
Suffering from food and drug allergies	0.4	65	26
Interval between receiving a COVID-19 vaccine and participating in this study	0.54	66	26
Education level	0.75	65	26
Number of doses **^	0.8	65	26
Experiencing COVID-19 vaccine hesitancy and related fears before vaccination	0.89	65	24
Gender**^	1.05	64	23
Time of breakthrough infection	1.16	63	21
Age **^	1.55	63	21
Suffering from chronic diseases	1.67	62	18
Type of COVID-19 vaccine **^	2.15	62	18
Country **^	2.24	60	9
Critical features		64	24
**Sleepiness and laziness**	**FI**	**A**	**C**
Being smoker	0.09	63	26
Experiencing vaccine breakthrough COVID-19 infection	0.17	63	26
Being healthcare worker	0.18	63	25
Number of doses	0.24	63	25
Suffering from food and drug allergies	0.46	63	25
Interval between receiving a COVID-19 vaccine and participating in this study	0.57	62	24

Suffering from food and drug allergies	0.2	74	22
Being smoker	0.31	74	22
Number of doses **^	0.34	74	22
Interval between receiving a COVID-19 vaccine and participating in this study	0.56	73	20
Gender	0.71	73	19
Experiencing vaccine breakthrough COVID-19 infection **^	0.78	74	19
Education level	0.91	73	17
Age	1.08	73	17
Experiencing COVID-19 vaccine hesitancy and related fears before vaccination **^	1.08	73	17
Time of breakthrough infection **^	1.67	73	15
Suffering from chronic diseases **^	1.69	72	13
Country**^	2.12	73	11
Type of COVID-19 vaccine **^	2.44	73	4
Critical features		73	19

FI, feature importance; A, accuracy; C, Cohen's value .

Education level	0.64	62	24
Time of breakthrough infection	0.64	62	23
Experiencing COVID-19 infection before receiving any vaccine dose	0.75	63	25
Experiencing COVID-19 vaccine hesitancy and related fears before vaccination **^	1.2	63	24
Type of COVID-19 vaccine	1.65	61	21
Diseases	1.69	60	20
Gender **^	1.81	60	20
Age	1.83	58	14
Country **^	2.03	58	14
Critical features		61	21
