# Supplementary material for: Reported Adverse Effects and Attitudes among Arab Populations Following COVID-19 Vaccination: A Large-Scale Multinational Study Implementing Machine Learning Tools in Predicting Post-Vaccination Adverse Effects Based on Predisposing Factors
Source: Vaccines (Basel). 2022 Feb 26;10(3):366. doi: 10.3390/vaccines10030366 (PMC8955470; doi:10.3390/vaccines10030366)
Supplement: Supplementary file 1 [file vaccines-10-00366-s001.zip › Survey tool-Arab.pdf]

vaccines

Survey of Side Effects and Perceptions Following COVID-19 Vaccination in the Arab World

- This survey aims to study the side effects appeared after receiving COVID-19 vaccines in the Arab world.
- This survey aims to study the perceptions of Arab people who received COVID-19 vaccines.
- This survey targets everyone received the first or second dose of any COVID-19 vaccines in the Arab world (regardless of the ethnicity, nationality, age, educational level...etc.).
- Elderly participants can ask a trusted person to assist, or to answer on behalf of them if necessary.
- This survey does not include the names or identifying information of participants.

SECTION 1: Participant consent

By participating in this study, you are contributing in the awareness-raising efforts about the safety and protection of COVID-19 vaccines, and thus you will help others to protect themselves and to refute any false rumors about these vaccines.

Do you agree to participate in this study?

- ☐ Yes
- ☐ No

SECTION 2: Participant information

1. Gender:

- ☐ Male
- ☐ Female

2. Age category (years):

- ☐ Below 20
- ☐ 20 – 39

- ☐ 40 – 59
- ☐ 60 years or above

3. Educational level:

- ☐ High school or below
- ☐ College or undergraduate
- ☐ Postgraduate

4. Are you a healthcare worker?

- ☐ Yes
- ☐ No

5. Country of residence:

- ☐ Algeria
- ☐ Bahrain
- ☐ Comoros
- ☐ Djibouti
- ☐ Egypt
- ☐ Iraq
- ☐ Jordan
- ☐ Kuwait
- ☐ Lebanon
- ☐ Libya
- ☐ Mauritania
- ☐ Morocco
- ☐ Oman
- ☐ Palestine
- ☐ Qatar
- ☐ Saudi Arabia
- ☐ Somalia
- ☐ Sudan
- ☐ Syria
- ☐ Tunisia
- ☐ United Arab Emirates

- Yemen

6. Are you suffering from any of chronic diseases?

(*You can select more than one choice*)

- No
- Diabetes mellitus
- Hypertension
- Cardiovascular diseases
- Respiratory diseases
- Obesity
- Joint inflammations
- Autoimmune diseases
- Thyroid disorders
- Cancer
- Other diseases (please specify): ……………………………………………………

7. Are you a smoker (cigarettes or shisha)?

- Yes
- No

8. Are you suffering from an allergy to any types of foods or medicines?

- Yes
- No

9. Have you been infected with COVID-19 before vaccination?

- Yes
- No

10. Did you feel scared to receive a COVID-19 vaccine before vaccination?

- Yes
- No

11. Before vaccination, which type of COVID-19 vaccines did you prefer?

- AstraZeneca/Oxford

- Pfizer-BioNTech
- Sinopharm
- Johnson & Johnson
- Moderna
- Sputnik V
- Covaxin
- I have no preference

12. How did you know about COVID-19 vaccines?

- Government-owned media platforms
- Social media platforms
- Friends and relatives
- Scientific and medical websites
- I have no information

13. Which type of COVID-19 vaccines have you received?

- AstraZeneca/Oxford
- Pfizer-BioNTech
- Sinopharm
- Johnson & Johnson
- Moderna
- Sputnik V
- Covaxin

14. How many doses have you received so far?

- Single dose
- Two doses

15. Interval between receiving a COVID-19 vaccine and participating in this study:

- Up to 3 weeks
- 3 – 8 weeks
- More than 8 weeks

16. Have you got infected with COVID-19 after vaccination (vaccine breakthrough infection)?

- ☐ Yes (answer question 17)
- ☐ No (move to question 18)

17. If you experienced a COVID-19 vaccine breakthrough infection, please specify the time after vaccination?

- ☐ During the first week
- ☐ Between one – three weeks
- ☐ More than three weeks

18. Have you noticed any side effects following COVID-19 vaccination?

- ☐ No side effects at all (*submit your answers*)
- ☐ Yes, mild side effects (*complete the next section*)
- ☐ Yes, moderate side effects (*complete the next section*)
- ☐ Yes, severe side effects (*complete the next section*)

19. Do you think that COVID-19 vaccines are safe in the long term?

- ☐ Yes
- ☐ No

20. Do you feel more reassured after vaccination?

- ☐ Yes
- ☐ No

21. Do you think that the following of sterilization and social distance measures, as well as wearing medical face masks, is still necessary after vaccination?

- ☐ Yes
- ☐ No

22. Are you monitoring your vital signs more frequent after vaccination?

- ☐ Yes
- ☐ No

23. Do you advice others to get vaccinated for COVID-19?

- ☐ Yes

☐ No

<u>SECTION 3: Side effects recorded after vaccination</u>

(*Please note that these side effects should be appeared suddenly and without known causes*).

24. Have you felt tiredness and fatigue?

☐ Yes

☐ No

25. Have you experienced decreased sleep quality?

☐ Yes

☐ No

26. Have you felt a fever?

☐ Yes

☐ No

27. Have you felt a headache?

☐ Yes

☐ No

28. Have you experienced haziness or lack-of-clarity in your eyesight?

☐ Yes

☐ No

29. Have you experienced pain or swelling at the injection site?

☐ Yes

☐ No

30. Have you felt joints pain?

☐ Yes

☐ No

31. Have you experienced swollen ankles and feet?

- Yes
- No

32. Have you felt muscle pain (myalgia)?

- Yes
- No

33. Have you felt nausea?

- Yes
- No

34. Have you felt abdominal pain?

- Yes
- No

35. Have you experienced diarrhea?

- Yes
- No

36. Have you experienced vomiting?

- Yes
- No

37. Have you noticed any bruises on your body?

- Yes
- No

38. Have you experienced bleeding gums?

- Yes
- No

39. Have you experienced a nosebleed?

- Yes
- No

40. Have you felt chills?

- Yes
- No

41. Have you experienced irritation and allergic skin reactions, or itchy skin?

- Yes
- No

42. Have you noticed that your body sweats for no reason?

- Yes
- No

43. Have you felt cold, numbness and tingling in limbs?

- Yes
- No

44. Have you felt dizzy?

- Yes
- No

45. Have you felt a clogged nose?

- Yes
- No

46. Have you felt a runny nose?

- Yes
- No

47. Have you felt dyspnea?

- Yes
- No

48. Have you felt chest pain?

- Yes

- No

49. Have you felt over sleepiness or laziness?

- Yes
- No

50. Have you felt faster or irregular heartbeats?

- Yes
- No

51. Have you experienced an increase or decrease in blood pressure?

- Yes
- No

52. Have you felt a sore or dry throat?

- Yes
- No

53. Have you experienced a cough?

- Yes
- No

54. How soon did the side effects appear after injection with a COVID-19 vaccine?

- Up to 4 hours
- 5 to 12 hours
- 13 to 24 hours
- 24 hours or more

55. How long did the side effects last?

- Less than one day
- 1 to 3 days
- 4 to 7 days
- More than 7 days

56. Please write down any other side affects you have experienced:

(*Optional*)

…………………………………………………………………………………………………….................................

....

57. How did you act to relieve the side effects that appeared after vaccination?

- ☐ I took a rest at home (*submit your answers*)
- ☐ I took painkillers while staying rested at home (*submit your answers*)
- ☐ I went to a doctor's clinic, but there was no need for hospitalization (*answer the next section*)
- ☐ I have been admitted to a hospital, and I received the required healthcare services (*answer the next question*)

SECTION 4: After visiting a doctor or hospital

58. Please write down those side effects caused a visit to doctor or hospitalization:

…………………………………………………………………………………………………….................................

....

59. Have you been diagnosed with any types of thrombosis (blood clots)?

- ☐ Yes
- ☐ No

60. Have you been diagnosed with low platelet count (thrombocytopenia)?

- ☐ Yes
- ☐ No
